# The Important Roles of Steroid Sulfatase and Sulfotransferases in Gynecological Diseases

**DOI:** 10.3389/fphar.2016.00030

**Published:** 2016-02-18

**Authors:** Tea Lanišnik Rižner

**Affiliations:** Faculty of Medicine, Institute of Biochemistry, University of LjubljanaLjubljana, Slovenia

**Keywords:** endometrial cancer, endometriosis, adenomyosis, uterine fibroids, estrogen formation, estrone sulfate, dehydroepiandrosterone sulfate

## Abstract

Gynecological diseases such as endometriosis, adenomyosis and uterine fibroids, and gynecological cancers including endometrial cancer and ovarian cancer, affect a large proportion of women. These diseases are estrogen dependent, and their progression often depends on local estrogen formation. In peripheral tissues, estrogens can be formed from the inactive precursors dehydroepiandrosterone sulfate and estrone sulfate. Sulfatase and sulfotransferases have pivotal roles in these processes, where sulfatase hydrolyzes estrone sulfate to estrone, and dehydroepiandrosterone sulfate to dehydroepiandrosterone, and sulfotransferases catalyze the reverse reactions. Further activation of estrone to the most potent estrogen, estradiol, is catalyzed by 17-ketosteroid reductases, while estradiol can also be formed from dehydroepiandrosterone by the sequential actions of 3β-hydroxysteroid dehydrogenase-Δ^4^-isomerase, aromatase, and 17-ketosteroid reductase. This review introduces the sulfatase and sulfotransferase enzymes, in terms of their structures and reaction mechanisms, and the regulation and different transcripts of their genes, together with the importance of their currently known single nucleotide polymorphisms. Data on expression of sulfatase and sulfotransferases in gynecological diseases are also reviewed. There are often unchanged mRNA and protein levels in diseased tissue, with higher sulfatase activities in cancerous endometrium, ovarian cancer cell lines, and adenomyosis. This can be indicative of a disturbed balance between the sulfatase and sulfotransferases enzymes, defining the potential for sulfatase as a drug target for treatment of gynecological diseases. Finally, clinical trials with sulfatase inhibitors are discussed, where two inhibitors have already concluded phase II trials, although so far with no convincing clinical outcomes for patients with endometrial cancer and endometriosis.

## Steroid sulfatase

**Figure 1:**
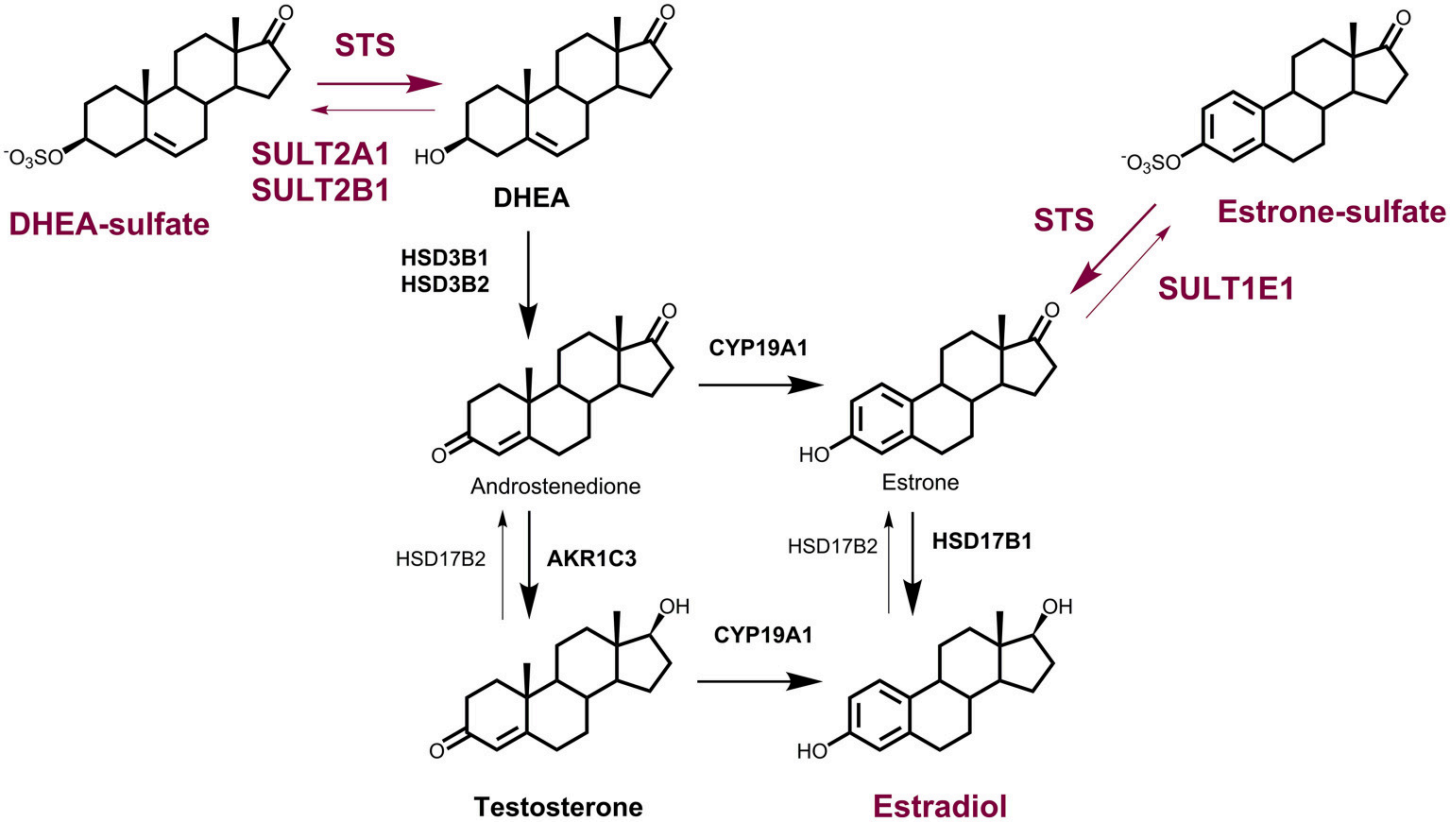
**The roles of the STS and SULT enzymes in local estrogen biosynthesis**. Synthesis of E2 from DHEA-S by the action of steroid sulfatase (STS), 3β-hydroxysteroid dehydrogenase-Δ^4^-isomerase (HSD3B1, HSD3B2), aromatase (CYP19A1) and 17-ketosteroid reductase (HSD17B1) or aldo-keto reductase 1C3 (AKR1C3) and CYP19A1; and from E1-S by the action of STS and HSD17B1. Sulfotransferases 2A1 (SULT2A1) and SULT2B1 catalyze the conjugation of DHEA, and SULT1E1 catalyzes the conjugation of E1. Oxidation of E2 to E1 is catalyzed by HSD17B2.

The formation and hydrolysis of steroid sulfates by the steroid sulfotransferase (SULT) and steroid sulfatase (STS) enzymes, respectively, represent important mechanisms in the regulation of the biological activities of many steroid hormones (Purohit et al., 1998). STS (E.C. 3.1.6.2.) hydrolyzes estrone sulfate (E1-S) and dehydroepiandrosterone sulfate (DHEA-S) to E1 and DHEA, respectively (Figure 1), as well as cholesterol sulfate and pregnenolone sulfate to their corresponding unconjugated forms. The sulfatase protein family includes 17 different human sulfatases, where only STS act on steroid sulfates (Mueller et al., 2015).

**Figure 2:**
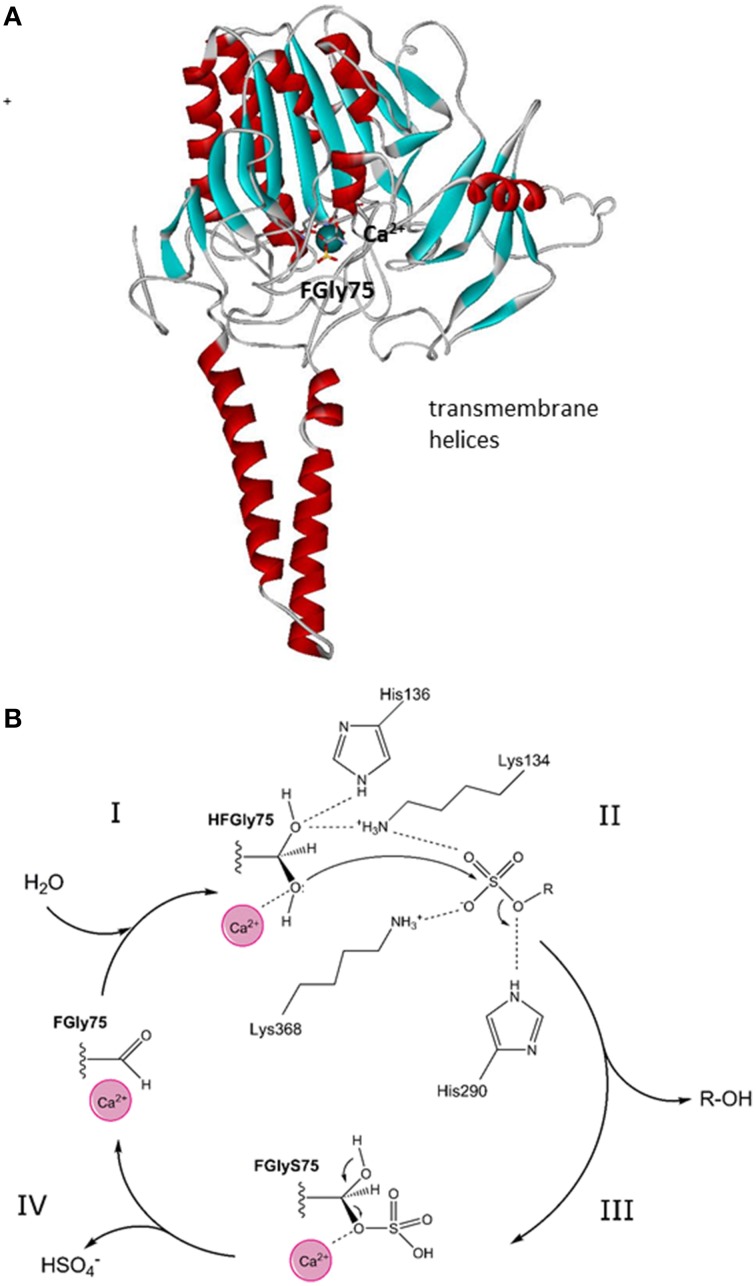
**Tertiary structure and reaction mechanism of the human STS enzyme. (A)** Structure of the human STS enzyme (pdb 1P49), with transmembrane helices and the globular domain with the active site with cofactor Ca^2+^. **(B)** The reaction mechanism of the STS enzyme and the roles of the catalytical amino-acid residues His136, Lys134, His290, and Lys368. FGly, formylglycine; HFGly, hydroxyformylglycine; HFGlyS, hydroxyformylglycine sulfate. The scheme was adopted from Ghosh (2007).

STS has been found in the membranes of the endoplasmic reticulum and was purified from human placenta (Hernandez-Guzman et al., 2001). It is a monomer with a molecular mass of 63 kDa, an N-terminal signal peptide of 21–23 amino acids, and four potential and two functional (i.e., Asn47, Asn259) glycosylation sites (Reed et al., 2005). Purified STS has been crystallized, and its structure has been defined at 2.6-Å resolution (pdb code 1P49; Hernandez-Guzman et al., 2001, 2003). The three-dimensional structure of STS shows a globular polar domain with the catalytic site, and the putative transmembrane domain that consists of two antiparallel hydrophobic alpha helices (Hernandez-Guzman et al., 2003) (Figure 2A). STS includes Ca^2+^ as a cofactor and 10 catalytically important amino acid residues: Arg35, Arg36, ARG78, Arg342, Lys134, Lys368, His136, His290, Gln343 and a formylglycine (FGly75). This posttranslational modification of Cys75 to FGly75 is mediated by the FGly-generating enzyme encoded by *sulfatase-modifying factor 1* (Preusser-Kunze et al., 2005).

STS catalyzes the hydrolysis of the sulfate moiety in a four-step mechanism. According to Ghosh (2007), these steps comprise: (1) activation of FGly75 by a water molecule; (2) nucleophilic attack of hydroxy-FGly on the sulfur atom of the substrate (i.e., E1-S, DHEA-S), which is facilitated by Ca^2+^; (3) release of the free hydroxy-product (i.e., E1, DHEA); and finally (4) release of ${\text{HSO}}_{4}^{-}$ and regeneration of FGly (Figure 2B). As a sulfate moiety covalently linked to hydroxy-FGly has been observed in the crystal structure of STS, this suggests that the sulfated form of hydroxy-FGly is the resting state for human STS (Ghosh, 2007). Kinetics characterization has revealed that purified STS can hydrolyze DHEA-S and E1-S with μM Km values (Hernandez-Guzman et al., 2001; Table 1).

**Table 1:** **Kinetics characteristics of the STS and SULT enzymes**.

**Enzyme**	**Substrate**	**K_m_**	**v_max_**	**v_max_/K_m_**	**Ki**	**References**
STS	DHEA-S	9.6 μM	113.4 μmol/h/mg	11.9		Hernandez-Guzman et al., 2001^a^
	E1-S	72.8 μM	573 μmol/h/mg	7.9		Hernandez-Guzman et al., 2001^a^
SULT1E1	E1	110 ± 100 nM	4.1 ± 0.1 nmol/h/mg	0.04		Adjei et al., 2003^b^
	E2	29 ±10 nM	6.9 ± 0.1 nmol/h/mg	0.24		Adjei et al., 2003^b^
SULT2A1	DHEA	2.5 ± 0.7 μM	13.6 ± 2.5 μmol/h/mg	5.4	6.1 μM	Lu et al., 2008^b^
SULT2B1a^*^	DHEA	2.3 ± 0.2 μM	0.3 ± 0.01 μmol/h/mg	0.13	48 μM	Geese and Raftogianis, 2001^b^
SULT2B1b^*^	DHEA	4.4 ± 0.6 μM	0.1 ± 0.01 μmol/h/mg	0.02	22 μM	Geese and Raftogianis, 2001^b^

a *Purified STS from human placenta*.

b *Purified recombinant human enzymes, mean ± SEM values are shown*;

* *SULT2B1a and SULT2B1b are two isoforms*.

The *STS* gene has been localized to the X chromosome (Xp22.31), and it spans 146 kb, includes 10 exons, and encodes a protein of 583 amino acids (Reed et al., 2005). Although eight tissue-specific *STS* transcripts have been identified (Nardi et al., 2009), only six are currently included in the gene database: isozyme S, and variants X1 to X5 (http://www.ncbi.nlm.nih.gov/gene). *STS* transcripts have 235 coding genetic variants (cSNPs) in the db SNP database (http://www.ncbi.nlm.nih.gov/SNP; September 2015), although only one of these cSNPs, Val307Ile, has a minor allele frequency (MAF) >0.01 (0.0172) (Table 2). SNPs have been reported in the promoter region and in introns and exons of the *STS* gene (Brookes et al., 2010; Matsumoto et al., 2010, 2013). Seven SNPs have been identified in the Japanese population, including one SNP in the 5′-untranslated region (155G>A), five in the 5′-flanking region, and one cSNP, Val476Met, with a frequency of 0.014 (Matsumoto et al., 2010). For three specific SNPs, functional analysis has revealed significantly decreased (155A) and increased (−2837A, −1588C) transcriptional activities in a reporter gene assay in MCF7 cells, but showed no effects at the protein and DHEAS STS activity levels for Val476Met variant (Matsumoto et al., 2013).

**Table 2:** **Different transcripts and genetic variants of the *STS* and *SULT* genes**.

**Gene**	**Gene**	**Transcript**	**Size**	**Protein**	**gSNP**	**cSNP**		
	**ID**	**information**	**(bp)**	**(aa)**		**Total**	**SYN**	**MAF** >**0.01**
STS	412	Isozyme SNM_000351.4	6377	583	4838	231	93	Val307Ile, 0.0172
		Variant X5XM_011545518	6340	578	3772	231	93	Val307Ile, 0.0172
		Variant X4XM_011545517	6215	578	3782	231	93	Val307Ile, 0.0172
		Variant X3XM_011545516	6662	590	7324	232	93	Val307Ile, 0.0172
		Variant X2XM_011545515	7336	590	7365	235	93	Val307Ile, 0.0172
		Variant X1XM_005274511	6441	723	7318	232	93	Val307Ile, 0.0172
SULT1E1	6783	Variant X2XM_011532210	723	181	911	88	23	–
		Variant X1XM_011532209	1697	294	1337	156	42	–
		NM_005420	1805	294	1300	156	42	–
SULT2A1	6822	NM_003167	1987	285	1207	163	48	Ala63Pro, 0.0240 Ala261Thr, 0.0407
SULT2B1	6820	Variant 2NM_177973	1228	365	3190	231	88	Arg33Gln, 0.0178
		Variant X1XM_005259182	802	221	935	153	57	–
		Variant 1 NM_004605	1281	350	1707	220	82	Arg18Gln, 0.0178

*gSNP, SNP in gene region; cSNP, SNP within coding region; SYN, synonymous; MAF, minor allele frequency; data from Gene and SNP databases (http://www.ncbi.nlm.nih.gov/gene, http://www.ncbi.nlm.nih.gov/SNP); as of September 2015*.

The loss of STS activity due to *STS* gene deletion or point mutations results in X-linked ichthyosis, a genetic skin disorder that affects 1 in 2000 to 1 in 6000 males and is characterized by typical scaling of the skin epidermis (Gelmetti and Ruggero, 2002). Interestingly, deletions and mutations have been identified mainly in the regions from 7 to 10 exon, which encodes the C-terminal substrate binding part (Gelmetti and Ruggero, 2002). This disease results from the inability to liberate cholesterol from cholesterol sulfate, and it affects the incorporation of cholesterol sulfate in the stratum corneum. It is associated with epidermal hyperplasia and the formation of additional layers of corneocytes, which leads to unacceptable sloughing of the skin epidermis (Maltais and Poirier, 2011; Elias et al., 2014). Kent et al. reported that boys with this genetic disorder have a significantly increased risk of attention deficit hyperactivity disorder and autism (Kent et al., 2008). Interestingly, associations between this disorder and several SNPs within the *STS* gene were also reported (Brookes et al., 2010), where the SNP rs12861247 was significantly associated with lower *STS* expression. In contrast to these studies, Stergiakouli et al. did not confirm these associations, although they observed associations between the SNP rs17268988 and symptoms of inattention (Stergiakouli et al., 2011).

STS is ubiquitously expressed, with its highest protein levels in placenta, and lower levels in breast, skin, liver, lung, ovary, adrenal gland, endometrium, brain, and some other tissues. The tissue levels vary across physiological and pathophysiological conditions, but our current understanding of the regulation of *STS* expression is still very limited (Nardi et al., 2009). STS expression is under the control of estrogens in breast cancer (Zaichuk et al., 2007), and it is down-regulated by GnRH agonists (Maitoko and Sasaki, 2004) and danazol (Fechner et al., 2007) in endometriotic tissue. In endometrium, no significant differences in STS expression have been reported at the mRNA level between the proliferative and secretory phases (Colette et al., 2013), while at the protein level, significant down-regulation was seen in the late secretory phase, compared to the menstrual phase (Dassen et al., 2007). STS expression can also be affected by the inflammatory cytokines: interleukin (IL)1α enhanced STS expression and mRNA levels in the SKOV3 epithelial ovarian cancer cell line, but not in the OSE normal ovarian surface epithelium cell line (Ren et al., 2015), and IL1β suppressed STS expression in endometrial stromal cells (Matsuoka et al., 2002). On the other hand, IL6 and tumor necrosis factor (TNF)α increased STS activity in a breast cancer cell line MCF7 not changing the mRNA levels, which suggested posttranslational modifications *via* STS glycosylation (Newman et al., 2000). In contrast, Sung et al. recently reported that in prostate cancer cell line PC-3 both, TNFα and insulin growth factor II regulate STS expression at the transcriptional level (Sung et al., 2013). STS expression is also regulated by micro (mi)RNA, as the overexpression of miR-142-3p significantly reduced STS mRNA levels in the St-T1b human endometrial stromal cell line (Kästingschäfer et al., 2015).

## Steroid sulfotransferases

The SULTs catalyze the transfer of a sulfuryl group from 3′-phosphoadenosine-5′-phosphosulfate (PAPS) to the target molecule. This conjugation is typically involved in inactivation/detoxification reactions for a variety of substrates, such as xenobiotics, therapeutic drugs, toxic compounds, chemical carcinogens, bile acids, hormones and neurotransmitters, and it is also an important pathway for hormonal regulation (Geese and Raftogianis, 2001). The superfamily of cytosolic human SULTs comprises 13 *SULT* genes and spans four families: SULT1 includes three phenol SULT subfamilies (SULT1A, SULT1B, SULT1C) and an estrogen SULT (SULT1E1); SULT2s catalyze sulfonation of the hydroxyl groups of steroids (SULT2A1, SULT2B1); and there are two orphan SULT families, SULT4 and SULT6 (SULT4A1, SULT6B1) (reviewed in Geese and Raftogianis, 2001; Lindsay et al., 2008). SULT2A1 catalyzes the sulfonation of DHEA, androgens and estrogens, while SULT2B1 catalyzes sulfonation of only DHEA (Lindsay et al., 2008). Although SULTs in general have broad substrate specificity, SULT1E1 (E.C. 2.8.2.4.) has significantly greater affinity for the estrogens than other SULTs, and it inactivates E1 and estradiol (E2) with nM Km values (Falany et al., 1995; Table 1). SULT2A1 (E.C. 2.8.2.14) and SULT2B1 (E.C. 2.8.2.2) both catalyze sulfation of DHEA, although SULT2A1 shows much higher catalytic efficiency (Lu et al., 2008). The SULTs need PAPS as a coenzyme, and thus SULT expression has to be accompanied by expression of at least one of the two PAPS synthase genes (Mueller et al., 2015).

**Figure 3:**
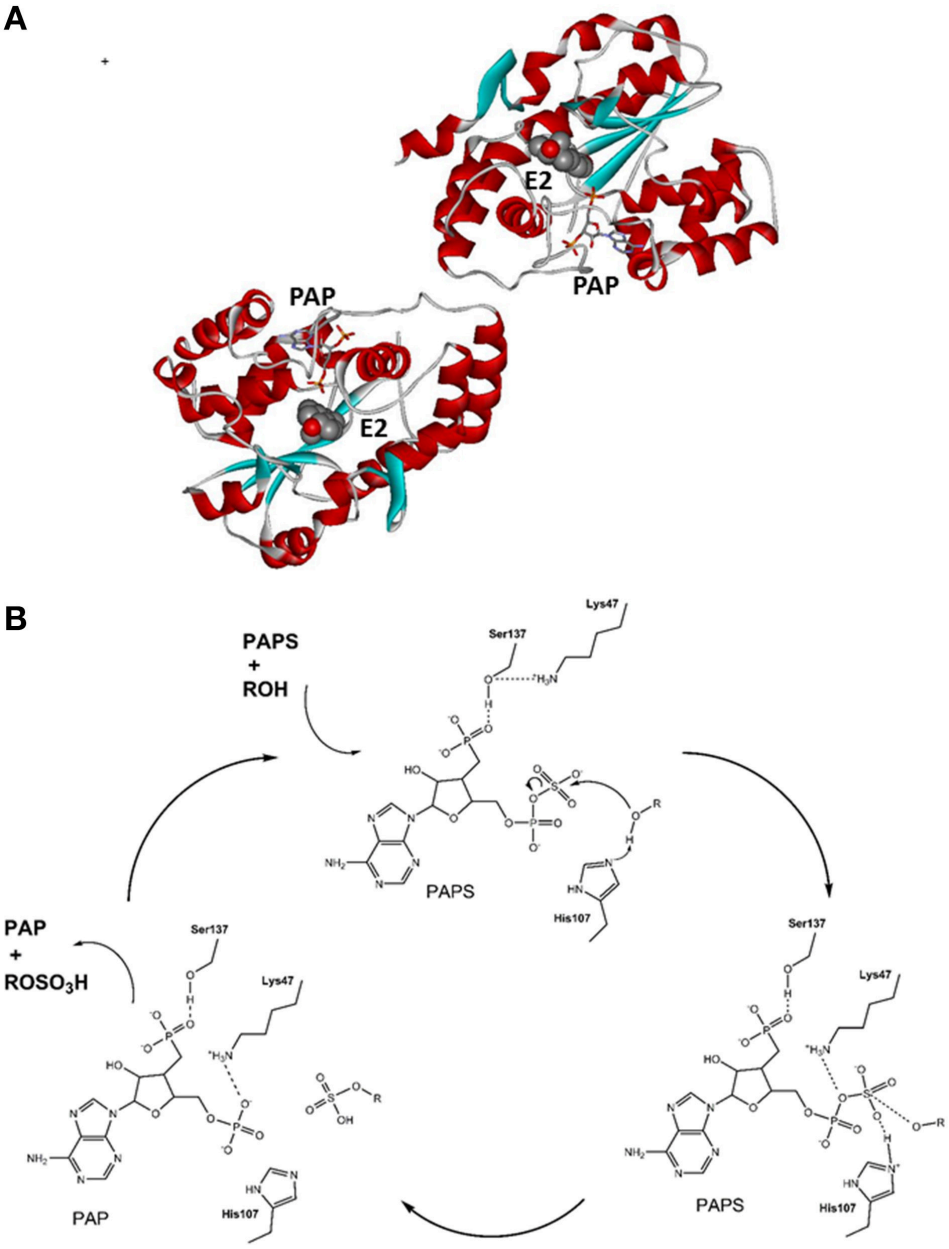
**Tertiary structure and reaction mechanism of the human SULT1E1 enzyme. (A)** Structure of the human SULT1E1 enzyme in complex with PAP and E2 (pdb 4JVL). **(B)** Reaction mechanism and the roles of catalytical amino-acid residues His107, Lys47, and Ser137. The scheme was adopted from Thomas and Potter (2013).

The *SULT1E1* gene has been localized to chromosome 4 (4q13.2), it has eight exons and a length of 20 kb, and it encodes a protein of 294 amino acids with a molecular mass of 35 kDa (Falany et al., 1995). Recombinant SULT1E1 has been crystalized, and the structure of the binary complex with PAPS (pdb code 1HY3; Pedersen et al., 2002) and several ternary complexes, including a complex with PAP and E2 (pdb code 4JVL; Gosavi et al., 2013), have been resolved. SULT1E1 is a dimer with an α/β motif that consists of five parallel β-strands surrounded by α-helices and a conserved α-helix across this structure (Pedersen et al., 2002) (Figure 3A). The structure of the SULT1E1–PAP–E2 complex has revealed that E2 binds to a mostly buried hydrophobic pocket with the sulfuryl acceptor 3′ hydroxyl within H-bonding distance of the proposed catalytic His107, and Lys10. The Phe80 and Phe141 are involved in the positioning of the steroidal substrate and have been suggested to function as a steric gate, thus defining substrate specificity (Gosavi et al., 2013). SULT1E1 has three catalytic amino-acid residues: Lys47, His107, and Ser137 (Negishi et al., 2001). The proposed reaction mechanism is as follows: upon binding of PAPS, Ser137 forms an H-bond with Lys47, thus prevents its interaction with the bridging oxygen in PAPS and its further hydrolysis; His107 attracts a proton from the substrate hydroxyl (i.e., of E2, DHEA), and enables nucleophilic attack at the sulfur atom in PAPS. In the next step, Lys47 interacts with the 5′ phosphate of PAPS, which help in dissociation of the sulfuryl group and in its transfer to the substrate. Finally, the reaction products, PAP and E1-S are released, which completes the catalytic cycle (Gamage et al., 2006; Tibbs et al., 2015) (Figure 3B). The crystallization and kinetics data have further suggested that the substrate inhibition that has been observed at high concentrations is a dead-end complex where both PAP and E2 are bound (Gosavi et al., 2013).

For the *SULT1E1* gene, in addition to the primary transcript, there are two variant transcripts (i.e., variants X1, X2) that encode proteins of 294 amino acids and 181 amino acids. Currently more than 150 cSNPs are included in the SNP database for *SULT1E1*, but none show MAF >0.01 (Table 2). Functional analysis has revealed decreased SULT1E1 activities for two nonsynonymous coding SNPs: Asp22Tyr and Ala32Val (Adjei et al., 2003). These cSNPs were found in African-American (Asp22Tyr) and Caucasian (Ala32Val) populations (Adjei et al., 2003). Additionally, the SNP rs6259 in the promoter region of *SULT1E1* has been associated with increased risk of endometrial cancer (Rebbeck et al., 2006; Hirata et al., 2008), and SNPs ^*^959 G>A and IVS4-1653 T>C with increased recurrence of endometrial cancer (Choi et al., 2005).

*SULT1E1* is expressed in liver, secretory endometrium, fetal liver, lung, and kidney (Coughtrie, 2002). Expression of *SULT1E1* is regulated by steroid hormones. In the normal endometrium, the highest expression of *SULT1E1* was found for the secretory phase, which is consistent with regulation by progesterone (Falany and Falany, 1996; Rubin et al., 1999; Dassen et al., 2007), although Colette et al. recently reported that *SULT1E1* expression is unchanged in the proliferative and secretory phases (Colette et al., 2013). In endometrial cancer cell line Ishikawa *SULT1E1* is induced by steroid drug tibolone via progesterone receptor (Falany and Falany, 2006), while in liver it is repressed by xenobiotic activators of the pregnane X receptor and aryl hydrocarbon receptor peroxisome proliferator, and activated via peroxisome proliferator activated receptor α and the liver X receptor (Duanmu et al., 2007). Furthermore, studies have implied that *SULT1E1* is also epigenetically regulated, as its expression is induced by the histone deacetylase inhibitor trichostatin A in MCF10A cells (Fu et al., 2010). SULT1E1 activity is also affected by ubiquitous pollutants, such as the polychlorinated biphenyls, which act as potent inhibitors with Ki values in the pM range, and thus they can exert their endocrine disrupting effects without binding to steroid hormone receptors (Kester et al., 2000).

The *SULT2A1* gene has been localized to chromosome 19 (19q13.3). It has six exons and a total length of 16 kb (Freimuth et al., 2004), with only one transcript known, which encodes a protein with 285 amino acids and a molecular mass of 34 kDa (Table 2). Several crystal structures of SULT2A1 binary complexes have been resolved (Tibbs et al., 2015), including complexes with DHEA (pdb code 1J99; Rehse et al., 2002) and PAPS (pdb code 4IFB). The complete kinetic mechanism of SULT2A1 has been defined, which revealed that the binding of DHEA and PAPS is random, while the rate-limiting step is nucleotide release. Here, the potent substrate inhibition with a Ki of 6.1 μM for DHEA can be explained by trapping PAP in a dead-end complex, which impedes the release of the nucleotide coenzyme (Wang et al., 2014).

*SULT2A1* is expressed in liver, adrenal gland, fetal adrenal, and fetal liver (Luu-The et al., 1995; Coughtrie, 2002). SULT2A1 has broad substrate specificity, as it can catalyze sulfonation of many hydroxysteroids, including DHEA, epiandrosterone, androsterone, testosterone, E2, cholesterol, various bile acids, pregnenolone, 17-ethinyl-E2 and cortisol (Lindsay et al., 2008). More than 160 cSNPs have been reported for *SULT2A1* (Table 2). Functional analysis has revealed that the nonsynonymous SNPs Met57Thr, Glu186Val, Ala63Pro, and Lys227Glu result in decreased SULT2A1 activity when expressed in COS-1 cells (Nagata and Yamazoe, 2000). Interestingly, the *SULT2A1* SNPs Ala63Pro, Lys227Glu and Ala261Thr have only been found in African-American populations, and these might explain the reported interethnic differences in SULT2A1 activity (Thomae et al., 2002; Hildebrandt et al., 2007). The *SULT2A1* SNP Ala261Thr has an allele frequency of 13% in African-American populations (reported MAF, 0.0407) and is located within the dimerization motif, where it prevents formation of SULT2A1 dimers. Surprisingly Ala261Thr still has 93% of the wild-type enzyme activity. Also Ala63Pro is quite common, with an allele frequency of 5% in African-American populations, and a reported MAF of 0.024 (Table 2).

The expression of *SULT2A1* is regulated at the transcriptional level by the constitutive androstane receptor and pregnane X receptor (Echchgadda et al., 2007). As recently shown in human hepatocytes and the HepG2 cell line, the *SULT2A1* gene is induced by E2 activation of estrogen receptor (ER)α via classical, direct binding to the estrogen response element, and via nonclassical, AP-1–mediated mechanisms (Li et al., 2014). *SULT2A1* appears to be regulated also at the epigenetic level, as shown by induction of *SULT2A1* expression after treatment of MCF7 cells with a histone deacetylase inhibitor (Fu et al., 2010). In the adrenal gland, the expression of *SULT2A1* depends on two transcription factors: steroidogenic factor 1 and GATA-6 (Saner et al., 2005).

The SULT2B1 isoforms SULT2B1a and SULT2B1b are products of alternative transcriptional initiation and mRNA splicing of the same gene, and are expressed in different tissues. SULT1B1a is expressed in colon, ovary, and fetal brain, and SULT2B1b in liver, colon, small intestine, placenta, ovary, uterus, and prostate (Geese and Raftogianis, 2001). SULT1B1a and SULT1B1b, which has an additional 23 N-terminal amino-acid residues, have been cloned and expressed in prokaryotic and eukaryotic systems, and purified and characterized (Geese and Raftogianis, 2001; Meloche and Falany, 2001). These enzymes catalyze sulfonation of 3β-hydroxysteroids, pregnenolone, 17α-hydroxypregnenolone and DHEA (Geese and Raftogianis, 2001). The structures of both SULT2B1a and SULT2B1b in binary complexes with PAP (pdb codes 1Q1Q, 1Q1Z, respectively Lee et al., 2003) have been resolved, as have two ternary complexes of SULT1Bb with PAP and pregnenolone (pdb code 1Q2O; Lee et al., 2003), and with PAP and DHEA (pdb code 1Q22, Lee et al., 2003; Tibbs et al., 2015).

The *SULT2B1* gene has been localized to chromosome 19 (19g13.3), and it comprises six exons with a total length of 48 kb (Geese and Raftogianis, 2001; Meloche and Falany, 2001; Freimuth et al., 2004). Three transcripts have been identified (variants 1, 2, X1) that encode proteins of 350, 365, and 221 amino acids (Table 2). Although more than 200 coding SNPs for *SULT2B1* are currently included in the SNP database, there is only one nonsynonymous SNP: Arg33Gln, with MAF >0.01 (Table 2). So far, only a few of these SNPs have been studied in more detail. Recently, Lévesque et al. (2014) reported that *SULT2B1* SNPs rs12460535, rs2665582, and rs10426628 are significantly associated with prostate cancer progression and hormone levels.

The SULT enzymes are known to show profound substrate inhibition, and SULT2B1 is not an exception. DHEA inhibits SULT2B1a and SULT2B1b with Ki values of 48 μM and 22 μM, respectively (Geese and Raftogianis, 2001). Not much is known about the regulation of *SULT2B1*. In normal endometrium, higher SULT2B1b mRNA levels have been seen in the mid-luteal phase, in agreement with progesterone regulation (Koizumi et al., 2010). In human endometrial stromal cells, the levels of SULT2B1b transcript were increased by cAMP or progesterone, while they were increased by cAMP or relaxin in endometrial epithelial cells, stimulating protein kinase A pathway (Koizumi et al., 2010). In prostate cancer cells, expression of SULT1B1b was shown to be regulated by vitamin D via heterodimer of vitamin D receptor and retinoid X receptor α (Seo et al., 2013).

## Local production of estrogens from DHEA-S and E1-S

STS and SULTs have pivotal roles in estrogen synthesis in peripheral tissues, and thus also in pre-receptor regulation of steroid hormone action. In premenopausal women, estrogens can be synthesized in the ovaries and in peripheral tissues (Labrie, 1991; Simpson, 2003). After menopause, when the ovaries cease to act, estrogens are formed only in the peripheral sites, which mainly include adipose tissue, but also bone, vascular endothelium, aortic smooth muscle cells, and brain (Simpson, 2003). Locally, estrogens can be formed from the inactive precursors of adrenal origin, DHEA-S, DHEA, and androstenedione, and of ovarian origin, DHEA and androstenedione, or from circulating E1-S (Figure 1). These steroid precursors are at relatively high concentrations in the blood of premenopausal and postmenopausal women, with DHEA-S at 3.4 ± 1.7 μM and 1.6 ± 1.0 μM, respectively (Labrie et al., 2006). DHEA and androstenedione are at 15.5 ± 7.6 nM and 3.4 ± 1.2 nM respectively, in premenopausal women, and 6.8 ± 4.1 nM and 1.4 ± 0.6 nM, respectively, in postmenopausal women (Labrie et al., 2006). Estrogens can thus be produced via the so-called aromatase pathway, from androstenedione and testosterone, by the actions of aromatase and 17-ketosteroid reductases (i.e., 17β-hydroxysteroid dehydrogenases; HSD17B), mainly HSD17B1. Estrogens can also be formed from DHEA-S by the actions of STS, 3β-hydroxysteroid dehydrogenases-Δ^4^-isomerase (HSD3B1, HSD3B2), aromatase (CYP19A1) and HSD17B or aldo-keto reductase 1C3 (AKR1C3) and CYP19A1, and from E1-S by the actions of STS and HSD17B (Rižner, 2013; Figure 1). The formation of E2 from E1-S is known as the STS pathway. E1-S is the most important estrogen in the peripheral blood, at 1.8 ± 1.1 nM and 0.6 ± 0.03 nM in premenopausal and postmenopausal women, respectively (Caron et al., 2009; Labrie et al., 2009). Studies in breast and endometrial cancers have shown that the STS pathway prevails over the aromatase pathway (Pasqualini et al., 1996; Chetrite et al., 2000; Purohit et al., 2011; Rižner, 2013). Higher E1-S, E1, and E2 plasma concentrations were observed for patients with breast cancer and endometrial cancer, compared to control women (Lépine et al., 2010; Tworoger et al., 2014) and high E1-S and E2 have been measured in breast cancer tissue (Chetrite et al., 2000), with high E2 and a high E2 to E1-S ratio reported in endometrial cancer tissue (Naitoh et al., 1989; Berstein et al., 2003). Most importantly, the levels of estrogens in breast and endometrial cancer tissue are 2- to 40-fold higher compared to plasma concentrations (Pasqualini et al., 1996; Berstein et al., 2003), further supporting intracrine formation and actions of estrogens.

DHEA-S and E1-S can thus serve as precursors for E2 formation after their translocation into cells via the transporter proteins of the organic anion-transporting polypeptide and organic anion-transporter families (Mueller et al., 2015). STS can then hydrolyze DHEA-S and E1-S to DHEA and E1, respectively, while the SULTs can catalyze the reverse reactions. SULT1E1 inactivates E1 and E2 to form the corresponding sulfates, and SULT2A1 and SULT2B1 conjugate DHEA (Raftogianis et al., 2000; Meloche and Falany, 2001; Gamage et al., 2006; Pasqualini, 2009). The sulfated steroids can then be excreeted from the cells via the ABC transporters (Mueller et al., 2015). The resulting increased uptake together with the decreased excretion of DHEA-S and E1-S, and the disturbed delicate balance between the STS and SULT enzymes, might thus lead to increased levels of estrogens, which are associated with hormone-dependent diseases, including several gynecological diseases.

## Sulfatase and sulfotransferases in gynecological diseases

Benign gynecological diseases such as endometriosis, adenomyosis, and uterine fibroids, and gynecological cancers such as endometrial cancer and ovarian cancer, affect a large proportion of women. The benign diseases are associated with infertility, and they can significantly decrease quality of life, while gynecological cancers comprise more than 10% of cancer-related deaths in women worldwide (Ferlay et al., 2013). These diseases are mainly hormone-dependent and thus rely on the local formation of active steroid hormones.

### Sulfatase and sulfotransferase in endometriosis

Endometriosis is a frequent benign gynecological disease that is characterized by the presence of endometrial tissue outside the uterine cavity. In the general population of women of reproductive age, the predicted prevalence of endometriosis is 6–10%, but the frequency increases to 30–50% in women with pain, infertility, or both (Guo and Wang, 2006; Rogers et al., 2013). Ectopic endometrial tissue can be found in different parts of the peritoneal cavity, where these locations define three different entities with different etiologies and pathogenesis: ovarian, peritoneal, and deep infiltrating endometriosis (Brosens and Benagiano, 2011). Endometriosis is an estrogen-dependent disease where endometriotic tissue proliferates in response to systemic estrogens and estrogens formed locally through the aromatase and the sulphatase pathways (Huhtinen et al., 2009; Rižner, 2009). However, the STS pathway appears to outperform the aromatase pathway, as the aromatase activity in endometriotic tissue is negligible when compared to STS activity (Purohit et al., 2008; Delvoux et al., 2009).

Data on expression of *STS* in endometriosis have remained relatively contradictory (Table 3). Although metabolism studies have revealed significantly lower STS activity in ectopic than eutopic endometrium of patients with ovarian endometriosis (Carlström et al., 1988), significantly higher levels of *STS* mRNA were seen for samples of ovarian endometriosis compared to eutopic endometrium from control patients (Smuc et al., 2007, 2009). Also, in peritoneal endometriosis, STS activity was lower in ectopic than matched eutopic endometrium, but it still correlated with severity of disease and was higher in moderate/severe disease vs. minimal/mild disease (Purohit et al., 2008). At the mRNA level, Collete et al. observed no significant differences between peritoneal endometriosis (ectopic) and control (eutopic) endometrial tissue but found higher levels in deep infiltrating endometriosis compared to ovarian endometriosis (Colette et al., 2013). Deep infiltrating endometriosis, Dassen et al. found no differences in *STS* expression between ectopic and eutopic endometrium of patients and the normal endometrium of the control group, while they found significantly lower protein levels of STS in epithelial cells of eutopic endometrium compared to normal endometrium (Dassen et al., 2007). Delvoux et al. reported unchanged STS activity in samples from all three types of endometriosis (Delvoux et al., 2009). However, Purohit et al. reported that STS activity in peritoneal ectopic implants and matched eutopic endometrium was higher than aromatase activity, which thus suggested that the STS pathway is important in estrogen formation and that STS inhibitors might be useful for treatment of endometriosis (Purohit et al., 2008; Delvoux et al., 2009).

**Table 3:** **Expression of STS and SULTs in endometriosis**.

**Gene**	**Level**	**Regulation**	**References**
*STS*	mRNA	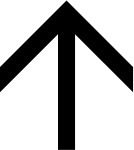 OE/control endometrium	Smuc et al., 2007, 2009
		≈ DIE/eutopic/normal endometrium	Dassen et al., 2007
		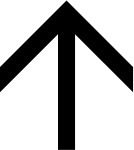 DIE/OE; ≈ PE/OE/eutopic endometrium	Colette et al., 2013
	Protein	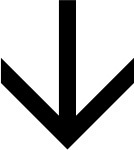 eutopic/normal endometrium	Dassen et al., 2007
		≈ ectopic/eutopic endometrium	Colette et al., 2013
	Activity (E1-S)	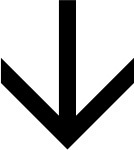 ectopic/eutopic	Carlström et al., 1988
		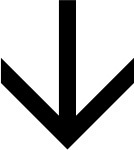 PE/eutopic endometrium	Purohit et al., 2008
		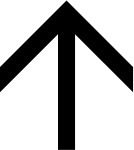 moderate/severe/minimal/mild	Purohit et al., 2008
		≈ ectopic/eutopic/control endometrium	Delvoux et al., 2009
*SULT1E1*	mRNA	≈ OE/control endometrium	Smuc et al., 2007, 2009
		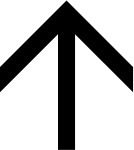 DIE/normal endometrium	Dassen et al., 2007
		≈ DIE, OE, PE/eutopic endometrium	Colette et al., 2013
		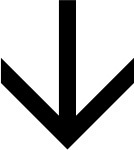 OE/normal endometrium	Hevir et al., 2013
		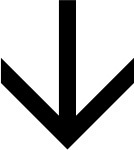 OE/eutopic endometrium	Borghese et al., 2008
	Protein	≈ OE, PE/eutopic/control endometrium	Hudelist et al., 2007
*SULT2B1*	mRNA	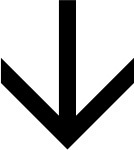 OE/normal endometrium	Hevir et al., 2013

*OE, ovarian endometriosis; PE, peritoneal endometriosis; DIE, deep infiltrating endometriosis*.

In addition to STS, SULTs should also be taken into account when evaluating the importance of the STS pathway, as both of these enzymes affect the subtle balance between estrogen activation (E1-S hydrolysis) and estrogen inactivation (E1, E2 sulfation). Expression of *SULT*s has been investigated in several studies (Table 3). In ectopic tissue of patients with ovarian, peritoneal, and deep infiltrating endometriosis, no significant differences in the expression of *SULT1E1* was seen at the mRNA level (Smuc et al., 2007, 2009; Colette et al., 2013), while there were significantly higher mRNA levels in ectopic endometrium of deep infiltrating endometriosis, when compared to the normal endometrium (Dassen et al., 2007). On the other hand, Borghese et al. and Hevir et al. found decreased mRNA levels in ovarian endometriosis vs. control eutopic endometrium from patients (Borghese et al., 2008) and control normal endometrium (Hevir et al., 2013), respectively. At the protein level, Hudelist et al. saw no differences between ovarian and peritoneal endometriotic lesions and eutopic endometrium (Hudelist et al., 2007). The expression of *SULT2A1* has not been studied in this context, but the expression of *SULT2B1* at the mRNA level was decreased in ovarian endometriosis compared to normal endometrium (Hevir et al., 2013); which implies that DHEA-S may serve as a precursor steroid in this ectopic tissue.

Data on the expression of the *STS* and *SULT* genes at the mRNA, protein and enzymatic activity levels in endometriosis tissue vs. control tissue are contradictory and do not fully support the importance of the sulfatase pathway. As different types of endometriosis show variations in the expression of these genes, it seems that the sulfatase may have roles primarily in ovarian endometriosis. Furthermore, the majority of studies have compared endometriotic tissue with eutopic endometrium of the same patient, which might not be the optimal study design. Nowadays, it is generally accepted that eutopic endometrium of endometriosis patients has already undergone pathological transformations, and that the gene expression profile in this tissue can be used as a biomarker of endometriosis (Burney et al., 2007).

### Sulfatase and sulfotransferase in adenomyosis

Adenomyosis is a benign gynecological disease that is defined as the presence of endometrial glands and stroma within the muscular layer of the uterus (i.e., myometrial tissue) (Kitawaki, 2006; Graziano et al., 2015). The symptoms that appear in two-thirds of patients with adenomyosis are dysmenorrhea, metrorrhagia, chronic pelvic pain, dyspareunia, and infertility. The prevalence of adenomyosis varies among studies, with 5–70% reported (Graziano et al., 2015). Adenomyosis mainly affects women in late reproductive age and regresses after menopause (Kitawaki, 2006). It is thus considered an estrogen-dependent disease, and is often associated with other estrogen-dependent pathologies, such as endometriosis, leiomyoma, and hyperplasia, and also with endometrial cancer (Kitawaki, 2006). Furthermore, expression of aromatase in adenomyotic tissue is also implicative of estrogen dependency (Kitawaki et al., 1997). However, the STS pathway for the local formation of estrogens has been studied by only two groups (Table 4). Ezaki et al. observed increased STS protein levels in adenomyotic tissue vs. the basilar layer of normal endometrium (Ezaki et al., 2001), and Yamamoto et al. reported increased E1-S STS activity in adenomyosis, which was reduced by danazol treatment (Yamamoto et al., 1993). Although the involvement of estrogens in the pathophysiology of adenomyosis has not been sufficiently studied, and thus further investigations into estrogen biosynthesis and actions in this gynecological disease are warranted, the published data indicates importance of the sulfatase pathway.

**Table 4:** **Expression of STS in adenomyosis**.

**Gene**	**Level**	**Regulation**	**References**
*STS*	Protein	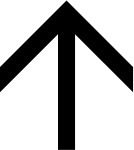 adenomyotic tissue/basilar layer of endometrium	Ezaki et al., 2001
	Activity (E1-S)	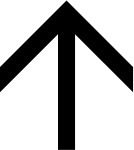 STS 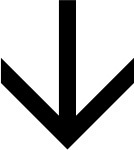 danazol treatment	Yamamoto et al., 1993

### Sulfatase and sulfotransferase in uterine fibroids

Uterine fibroids (leiomyomas; *myoma uteri* or *uterus myomatosus*) are benign tumors of the myometrium that occur in up to 70% of women of reproductive age (Bulun, 2013). However, the clinically significant symptoms that include heavy menstrual bleeding, anemia, difficulties with conceiving, and increased risk of miscarriage, are seen in one-third of the affected population (Bulun, 2013). Uterine fibroids appear after menarche and decline after menopause, which implicates the estrogens as the primary factor that drives their growth. Additionally, studies showed that estrogens are necessary, but not sufficient for proliferation and progesterone also has an important role in growth of leiomyomas. Estrogens act via estrogen receptor α and thus stimulate expression of progesterone receptors and further pro-proliferative action of progesterone (Moravek et al., 2015).

Based on PubMed searches, there has been only one report on the STS pathway in myoma tissue (Table 5). The Okada group examined E1-S hydrolysis and E1 sulfation in myoma tissue vs. myometrium, and they showed decreased STS and increased SULT activities (Yamamoto et al., 1990, 1993), thus suggesting that more estrogens can be formed in the surrounding myometrium than in the myoma tissue. In this way, estrogens might still act in a paracrine manner to provide myoma tissue with the mitogenic E2. As other studies showed that aromatase is upregulated, the local estrogen formation via the aromatase pathway probably has more important role. Furthermore, the effects of gonadotropin-releasing hormone analogs on the levels of estrogens and STS activity have also been examined in myoma where lower levels of estrogens (Pasqualini et al., 1990; van de Ven et al., 2002) and no significant effects on STS activity (van de Ven et al., 2002) were seen. So far the roles of the sulfatase pathway in uterine fibroids have not been thoroughly examined, which calls for further studies.

**Table 5:** **Expression of STS and SULT in myoma uteri**.

**Gene**	**Level**	**Regulation**	**References**
*STS*	Activity (E1-S)	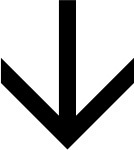 myoma vs. myometrium	Yamamoto et al., 1990
*SULT1E1*	Activity (E1)	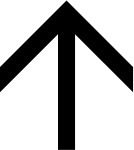 myoma vs. myometrium	Yamamoto et al., 1990

### Sulfatase and sulfotransferase in endometrial cancer

Endometrial cancer is the most common gynecological malignancy in the developed world. Worldwide, 319,605 new cases of endometrial cancer and 76,160 deaths were estimated for 2012 (Ferlay et al., 2013). Based on histopathology, the majority of endometrial cancers can be divided into two groups: type 1 with an endometrioid histology, which comprises 70–80% of all cases (Yeramian et al., 2013); and type 2 with serous papillary or clear-cell histology, which comprise 20% of all cases. Type 1 endometrial cancer is considered to be estrogen dependent, and type 2 not to be associated with hyper-estrogenic factors (Amant et al., 2005; Ryan et al., 2005). Development of type 1 endometrial cancer is usually explained by the unopposed estrogen hypothesis, which proposes that exposure to estrogens (of either endogenous or exogenous origins) that is not opposed by progesterone or synthetic progestins increases the mitotic activity of endometrial cells and the number of DNA replication errors. This can lead to somatic mutations that can result in malignant phenotypes (Henderson and Feigelson, 2000; Akhmedkhanov et al., 2001; Inoue, 2001; Sonoda and Barakat, 2006). In line with intracrine estrogen action the formation of estrogens has been detected in proliferative and secretory endometrium and in endometrial cancer tissues (Tseng et al., 1982). Additionally, increased E2 concentrations have been reported in cancerous endometrium of premenopausal and postmenopausal women with type 1 and type 2 endometrial cancer (Bonney et al., 1986; Berstein et al., 2003), and higher E1-S plasma concentrations were observed for endometrial cancer patients, compared to control healthy postmenopausal women (Jasonni et al., 1984).

Expression of STS and its activity in endometrial cancer have been evaluated in nine studies (Table 6). No significant differences in *STS* expression (Smuc and Rizner, 2009) and increased mRNA levels of *STS* (Lépine et al., 2010) have been reported in endometrial cancer vs. adjacent control tissue. At the cellular level, STS immunoreactivity in endometrial cancer tissue was higher compared to normal endometrium (Utsunomiya et al., 2004), which suggests increased availability of the biologically active estrogens. In line with this, immunohistochemical data and early metabolism studies have shown significantly higher E1-S STS activity in endometrial cancer tissue, compared to control endometrium (Adessi et al., 1984; Prost et al., 1984; Naitoh et al., 1989; Urabe et al., 1989). However, more recently, Tanaka et al. reported lower E1-S STS activity in cancerous tissue, compared to normal endometrium (Tanaka et al., 2003). This discordance might be explained by the different control groups or menopausal stages of the cases and controls. Increased DHEA-S STS activity has also been observed in endometrial cancer, compared to normal endometrium (Abulafia et al., 2009).

**Table 6:** **Expression of STS and SULTs in endometrial cancer**.

**Gene**	**Level**	**Regulation**	**References**
*STS*	mRNA	≈ Cancer/adjacent tissue; EC type 1	Smuc and Rizner, 2009
		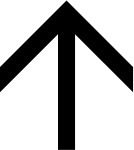 Cancer/adjacent tissue; EC type 1 (74%) and type 2 (26%)	Lépine et al., 2010
	Protein	86% EC	Utsunomiya et al., 2004
		82% Cancer weak to moderate/normal endometrium weak (glandular cells)	Human Protein Atlas
	Activity (E1-S)	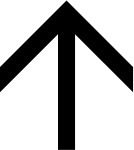 EC/normal endometrium	Adessi et al., 1984; Prost et al., 1984; Naitoh et al., 1989; Urabe et al., 1989
		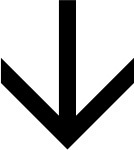 EC/normal endometrium	Tanaka et al., 2003
	DHEA-S	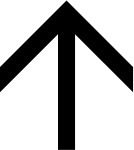 EC/normal endometrium	Abulafia et al., 2009
*SULT1E1*	mRNA	≈ Cancer/adjacent tissue; EC type 1	Hevir et al., 2011
		Borderline 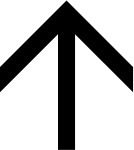 Cancer/adjacent tissue; EC type 1 (74%) and type 2 (26%)	Lépine et al., 2010
	Protein	22% EC	Utsunomiya et al., 2004
		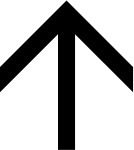 EC/adjacent endometrium	Xu et al., 2012
		Not detected in cancer	Human Protein Atlas
*SULT2B1*	mRNA	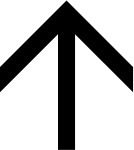 Cancer/adjacent tissue; EC type 1	Hevir et al., 2011
	Protein	Moderate staining in cancer	Human Protein Atlas

*EC, endometrial cancer*.

In cancerous and adjacent control endometrium, *SULT1E1* mRNA levels were reported to be unchanged or borderline significantly up-regulated (Lépine et al., 2010; Hevir et al., 2011; Table 6). The study by Lépine et al. (2010) included 26% type 2 endometrial cancer patients, which might explain these differences observed. At the cellular level, SULT1E1 immunoreactivity was diminished (Utsunomiya et al., 2004) or was not detected (www.proteinatlas.org; Uhlén et al., 2015) in endometrial cancer, compared to normal endometrium, while in other studies, increased SULT1E1 staining has been observed in cancerous endometrium, compared to adjacent control endometrium (Xu et al., 2012). These differences can be explained by different antibodies used. As *SULT2A1* is not expressed in either endometrial cancer or adjacent control endometrium (Rubin et al., 1999), SULT2B1 is the major DHEA-specific SULT enzyme in endometrial cancer, and increased *SULT2B1* expression has recently been reported in endometrial cancer, compared to adjacent control endometrium (Hevir et al., 2011). Also Human Protein Atlas data show medium to high staining of cancerous endometrium with two different anti-SULT2B1 antibodies (Uhlén et al., 2015), which further supports the conclusion that despite of increased DHEA-S STS activity, DHEA-S may not represent the steroid precursor for E2 formation in cancerous endometrium.

Although the expression of the *STS* and *SULT1E1* genes in diseased vs. control endometrium has differed between studies (Table 6), all of the published data show higher mRNA and protein levels for *STS* than *SULT1E1* in endometrial cancer tissue vs. control endometrium. The majority of the published data also shows increased E1-S STS activity in endometrial cancer, and thus supports E2 formation via the STS pathway.

### Sulfatase and sulfotransferase in ovarian cancer

Ovarian cancer is the sixth leading cause of cancer-related death in the developed world. Worldwide, there were 238,719 new cases and 151,905 deaths estimated for 2012 (Ferlay et al., 2013). Most patients are diagnosed when they show advanced stage disease with poor prognosis. Ovarian cancer is a heterogeneous disease that encompasses several types of tumors, with large differences in histopathological features and clinical behaviors. Epithelial ovarian cancers are the most frequent tumors, which account for around 98% of all cases. These can be defined into distinct diseases, as serous (75%), endometrioid (10%), clear-cell (5–10%), and mucinous (5–10%) ovarian cancers, which are usually grouped into low-grade or high-grade tumors (Guarneri et al., 2011). Nowadays, it is widely accepted that serous, mucinous, endometrioid, clear cell, and other histotypes of ovarian cancers represent molecularly and etiologically distinct diseases (Vaughan et al., 2011). The etiology of ovarian cancer can be explained by the “incessant ovulation theory” and the “gonadotropin theory” (Stadel, 1975; Fathalla, 2013). An increasing body of data suggests that ovarian cancer is estrogen dependent, although the role of estrogens in this disease has not yet been investigated thoroughly (Chura et al., 2009). Genetic susceptibility studies have associated SNPs in the *ESR2* gene that codes for ERβ with significantly increased risk of ovarian cancer (Lurie et al., 2011), and epidemiological studies (Women's Health Initiative and Million Women Studies: (Anderson et al., 2003; Beral et al., 2007) have also suggested that both estrogen only and estrogen–progestin hormone replacement therapies increase the risk of ovarian cancer (Modugno et al., 2012). However, the current lack of knowledge warrants further studies on the mechanisms of steroid hormone actions in ovarian cancer, followed by clinical trials that may also target estrogen actions.

The expression of STS has been studied at the mRNA and protein levels (Table 7). No significant differences were reported between epithelial ovarian carcinoma and ovarian surface epithelia tissue and primary cell cultures at the mRNA and protein levels (Ren et al., 2015). However, Okuda et al. detected STS staining in 70% of ovarian clear-cell adenocarcinoma, 33% of serous adenocarcinoma and 50% of mucinous adenocarcinoma, thus suggesting the importance of STS in this pathophysiology (Okuda et al., 2001). In contrast to these data, the Human Protein Atlas reports low to medium expression of STS in only two samples of mucinous adenocarcinoma out of 12 serous, mucinous and endometrioid ovarian cancer samples (Uhlén et al., 2015). The STS activity has been studied by two groups: Ren et al. reported higher activity in the SKOV-3 and PEO epithelial ovarian carcinoma cell lines vs. the OSE normal ovarian surface epithelium cell line (Ren et al., 2015), and Chura et al. reported correlations between increased STS activity and lower progression-free survival of patients (Chura et al., 2009).

**Table 7:** **Expression of STS and SULTs in ovarian cancer**.

**Gene**	**Level**	**Regulation**	**References**
*STS*	mRNA	≈ EOC/OSE	Ren et al., 2015
	Protein	Expressed EOC and OSE	Ren et al., 2015
		70% OCCA; 33% serous and 50% mucinous adenocarcinoma	Okuda et al., 2001
		17% Cancer low/moderate Normal ovary low (stromal cells)	Human Protein Atlas
	Activity (E1-S)	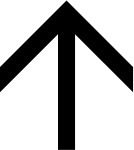 SKOV-3, PEO-1 vs. OSE	Ren et al., 2015
		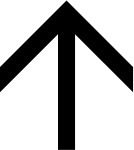 STS 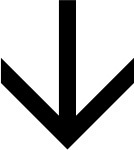 progression-free survival	Chura et al., 2009
*SULT1E1*	mRNA	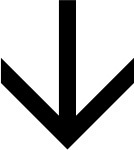 EOC/OSE	Ren et al., 2015
	Protein	Expressed EOC and OSE	Ren et al., 2015
		17% Cancer moderate/normal ND	Human Protein Atlas
	Activity (E1)	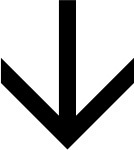 SKOV-3, PEO-1 vs. OSE	Ren et al., 2015
*SULT2A1*	Protein	17%-50% Cancer low/moderate Normal ovary/ND	Human Protein Atlas
*SULT2B1*	Protein	58%-100% Cancer moderate Normal ovary/weak	Human Protein Atlas

*EOC, epithelial ovarian carcinoma; OSE, ovarian surface epithelia; OCCA, ovarian clear-cell adenocarcinoma*.

The expression of the *SULT1E1* gene has only been investigated by one group (Ren et al., 2015; Table 7). They reported that *SULT1E1* expression was down-regulated in epithelial ovarian carcinoma vs. the OSE normal ovarian surface epithelium cell line, and also that SULT activity was lower in SKOV-3 and PEO cells vs. OSE cells. This all supports the importance of local estrogen formation via the STS pathway (Ren et al., 2015). Using three different anti-SULT1E1 antibodies, SULT1E1 was detected in two of 12 cores of ovarian cancer, with low to high staining observed mainly in mucinous ovarian cancer (Uhlén et al., 2015). With two polyclonal anti-SULT2A1 antibodies, SULT2A1 was detected in two to six of 12 cores, with low to medium intensity, while with three different anti-SULT2B1 antibodies, medium to high intensity staining was reported for SULT2B1 in seven to 12 of 12 tissue microarray cores (Uhlén et al., 2015). To date, there have been no other reports on the expression of SULT2A1 and SULT2B1 in ovarian cancer. Based on these data, DHEA-S cannot be excluded as a potential precursor molecule for local estrogen formation in ovarian cancer.

## Sulfatase as a target for treatment of gynecological disease

There is a plethora of data that supports the important role of STS in individual gynecological diseases, with disturbances reported for the balance between the STS and SULT enzymes. Although the observed STS mRNA and protein levels were rarely increased, higher STS activities have been seen in the majority of pathological tissues and in model cell lines, including cancerous endometrium (Adessi et al., 1984; Prost et al., 1984; Naitoh et al., 1989; Urabe et al., 1989; Day et al., 2009), ovarian cancer cell lines (Day et al., 2009; Ren et al., 2015), and adenomyosis (Yamamoto et al., 1993; Ezaki et al., 2001). This is in line with the higher catalytic efficiency of STS vs. SULT (Table 1), and in some cases, higher expression levels of *STS* (Tables 3, 6). Furthermore, in endometriosis, endometrial cancer, and ovarian cancer, the STS pathway has been shown to prevail over the aromatase pathway (Purohit et al., 2008; Fournier and Poirier, 2009; Hevir-Kene and Rižner, 2015; Ren et al., 2015), which substantiate the potential of STS as a drug target.

Lack of correlation between STS mRNA levels and STS activity imply that post-translational modifications might increase STS activity. Additionally, increased uptake and decreased excretion of the sulfated steroids might also be recognized as enhanced STS activity, which would explain why RNA levels are less informative (Newman et al., 2000). As not much is known about the transport of E1-S and DHEA-S across the plasma membrane in these diseased tissues, their uptake and excretion should be studied, as this will help us to better understand local estrogen formation and actions.

## Sulfatase inhibitors and clinical trials

The development of STS inhibitors started in the 1990s, and to date, this has led to two compounds that have entered clinical trials (Poirier, 2015). STS inhibitors have been developed by several research groups and several pharmaceutical companies, and the current status of this field has been a topic of several educative reviews in the last few years (Mostafa and Taylor, 2013; Sadozai, 2013; Thomas and Potter, 2013, 2015; Williams, 2013; Poirier, 2015). In general, the large number of STS inhibitors known to date can be divided into steroidal and nonsteroidal compounds, and further into sulfomoylated and nonsulfomoylated compounds (Maltais and Poirier, 2011). Among the steroidal compounds, estrogen O-sulfamates have been the most intensively studied in *in-vitro* and *in-vivo* models. These have included E1-3-O-sulfamate (EMATE) and E2-3-O-sulfamate (E2MATE), where EMATE was identified as the first irreversible STS inhibitor, although it was originally developed as a prodrug for estrogen replacement therapy (Sadozai, 2013; Thomas and Potter, 2015).

Nonsteroidal inhibitors have also been developed, and an irreversible STS inhibitor, 667 Coumate (also known as STX64 and BN83495, and more recently referred to as irosustat), shows higher potency than EMATE (IC_50_, 8 nM vs. 25 nM, at 20 μM E1-S, against STS from placental microsomes) (Woo et al., 2000). This compound has high bioavailability, which was explained by its sequestration within erythrocytes, where it binds to carbonic anhydrase II (IC_50_, 25 nM) (Ho et al., 2003). A plethora of EMATE and 667 Coumate derivatives have been developed in the last few years as 2nd and 3rd generation STS inhibitors (Sadozai, 2013; Thomas and Potter, 2015). These have included the steroidal compound KW-2581 that showed no estrogenic activity (Ishida et al., 2007), and STX213 and STX1938, which have shown greater potency than 667 Coumate (Foster et al., 2008b).

In addition to classical STS inhibitors, dual inhibitors that target STS and aromatase have also been developed (Sadozai, 2013), as well as compounds with dual actions as STS inhibitors and prodrugs of selective estrogen modulators (Sadozai, 2013). Here, the nonsteroidal dual aromatase–STS inhibitor STX681 has shown great potential for treatment of hormone-dependent breast cancer (Foster et al., 2008a), and the dual sulfamate STS inhibitor and antiestrogen SR16157 has also been studied (Rasmussen et al., 2007; Sadozai, 2013).

The published data imply that inhibitors of STS have the potential for treatment of individual gynecological diseases. In the case of endometriosis, STS activity correlates with the severity of this disease, and Purohit et al. reported that the STS inhibitor STX64 almost completely blocked STS activity *in vitro* in eutopic and ectopic tissue from patients with peritoneal endometriosis (Purohit et al., 2008). Furthermore, E2MATE inhibited STS activity in endometrial tissue *in vitro* and *in vivo* in animal models, including in mouse uterus, liver, leukocytes and endometriotic lesions (Colette et al., 2011). Also, the inhibitor STX64 has been investigated in intact mouse endometrial cancer xenografts and ovariectomized mouse endometrial cancer xenograft models, and in the latter case, STX64 showed significant inhibition after daily 1–10 mg/kg oral doses (Foster et al., 2008c), thus demonstrating the great potential of STS inhibitors as novel anticancer drugs. To the best of our knowledge, STS inhibitors have not yet been investigated in ovarian cancer, adenomyosis and myoma uteri, neither *in vitro* nor *in vivo*.

Several clinical studies have already been performed with STS inhibitors (Thomas and Potter, 2015). Irosustat (i.e., 667 Coumate) was the first STS inhibitor to enter phase I clinical trials, in post-menopausal women with ER-positive breast cancer (NCT01840488); (Stanway et al., 2006). Later, two phase II clinical studies were reported in breast cancer patients (ClinicalTrials.gov): the IPET trial was designed to look at the effects of irosustat using positron emission tomography scanning (Thomas and Potter, 2015), while the IRIS trial investigated the effects of combined treatment with irosustat and anastrozole (an aromatase inhibitor) (NCT01785992). Unfortunately no study data are publicly available for these trials.

Irosustat has also been investigated in ER-positive advanced/recurrent endometrial cancer in a phase II study (NCT00910091). In this study, irosustat was evaluated vs. progestin megestrol acetate in 73 patients, with median age 68 years (range, 37–85 years). Here, 36 patients were randomized to irosustat (40 mg/day), and 37 to megestrol acetate (160 mg/day) (Pautier et al., 2012). After 6 months of treatment, 36.1 and 56.8% of the patients treated with irosustat and megestrol acetate, respectively, were alive and without disease progression. Progression free survival was 16 and 32 weeks for irosustat and megestrol acetate, respectively. Thus, in patients with advanced and recurrent disease, irosustat showed much lower clinical benefit compared to progestin megestrol acetate, which questioned the suitability of STS inhibitors for treatment of endometrial cancer.

In a murine model, E2MATE reduced the weight of the endometriotic lesions, but had no effects on proliferation and apoptosis, or on STS expression (Colette et al., 2011), which led the Swiss-based biopharmaceutical company PregLem to investigate E2MATE in endometriosis patients. As described by Pohl et al. they first examined the effects of E2MATE in a phase I study (SAPHIR; EudraCT number: 2007-005662-12) in healthy women of reproductive age, through which they showed that E2MATE can inhibit STS in peripheral blood mononuclear cells, although it failed to inhibit STS in eutopic endometrium (Pohl et al., 2014). As a continuation, they investigated the effects of E2MATE (4 mg/week) and/or norethindrone acetate (NETA; 10 mg/day) in healthy nonpregnant women of reproductive age (i.e., 24–39 years). Twenty-four women were randomized to E2MATE, NETA or the combination E2MATE+NETA, for 4 weeks of treatment and 12 weeks of follow-up. In both the E2MATE and E2MATE+NETA groups, the STS activity in peripheral blood mononuclear cells and in the endometrium decreased by 90–91% and 89–96%, respectively, during treatment, and these inhibition levels remained high also 1 month after the treatments (Pohl et al., 2014). E2MATE and NETA have been further studied in endometriosis patients in a phase II study (EudraCT Number: 2011-005167-24), although at present, no results are publicly available.

## Conclusions and perspectives

The STS pathway is clearly implicated in local estrogen formation, and is thus also associated with enhanced estrogen actions. A vast amount of data supports an important role for STS in hormone-dependent diseases, including gynecological diseases like endometrial cancer, ovarian cancer, endometriosis, and adenomyosis, and indirectly also in uterine fibroids. However, the clinical studies that have evaluated STS inhibitors in gynecological diseases to date have not provided convincing data. Although the dual STS and aromatase inhibitors and dual action inhibitors/antiestrogens might provide better clinical performances compared to the classical STS inhibitors, these agents have not reached clinical studies to date. As the intricate local estrogen formation also depends on membrane transport of the sulfated steroids, this aspect needs to be investigated more in detail. This knowledge might contribute to a logical interpretation of the clinical studies to date, and might lead to the identification of novel drug targets and combined treatments that will target individual transporters and the STS enzyme.

## Author contributions

The author confirms being the sole contributor of this work and approved it for publication.

### Conflict of interest statement

The author declares that the research was conducted in the absence of any commercial or financial relationships that could be construed as a potential conflict of interest.

## References

[B1] Abulafia O. Lee Y. C. Wagreich A. Economos K. Serur E. Nacharaju V. L. (2009). Sulfatase activity in normal and neoplastic endometrium. Gynecol. Obstet. Invest. 67, 57–60. 10.1159/000161571 18843186

[B2] Adessi G. L. Prost O. Agnani G. Petitjean A. Burnod J. (1984). Estrone sulfatase activity in normal and abnormal endometrium. Arch. Gynecol. 236, 13–18. 10.1007/BF02114864 6508360

[B3] Adjei A. A. Thomae B. A. Prondzinski J. L. Eckloff B. W. Wieben E. D. Weinshilboum R. M. (2003). Human estrogen sulfotransferase (SULT1E1) pharmacogenomics: gene resequencing and functional genomics. Br. J. Pharmacol. 139, 1373–1382. 10.1038/sj.bjp.0705369 12922923 PMC1573968

[B4] Akhmedkhanov A. Zeleniuch-Jacquotte A. Toniolo P. (2001). Role of exogenous and endogenous hormones in endometrial cancer: review of the evidence and research perspectives. Ann. N.Y. Acad. Sci. 943, 296–315. 10.1111/j.1749-6632.2001.tb03811.x 11594550

[B5] Amant F. Moerman P. Neven P. Timmerman D. Van Limbergen E. Vergote I. (2005). Endometrial cancer. Lancet 366, 491–505. 10.1016/S0140-6736(05)67063-8 16084259

[B6] Anderson G. L. Judd H. L. Kaunitz A. M. Barad D. H. Beresford S. A. Pettinger M.. (2003). Effects of estrogen plus progestin on gynecologic cancers and associated diagnostic procedures: the Women's Health Initiative randomized trial. JAMA 290, 1739–1748. 10.1001/jama.290.13.1739 14519708

[B7] Beral V. Bull D. Green J. Reeves G. Collaborators M. W. S. (2007). Ovarian cancer and hormone replacement therapy in the Million Women Study. Lancet 369, 1703–1710. 10.1016/S0140-6736(07)60534-0 17512855

[B8] Berstein L. M. Tchernobrovkina A. E. Gamajunova V. B. Kovalevskij A. J. Vasilyev D. A. Chepik O. F.. (2003). Tumor estrogen content and clinico-morphological and endocrine features of endometrial cancer. J. Cancer Res. Clin. Oncol. 129, 245–249. 10.1007/s00432-003-0427-9 12695909 PMC12161952

[B9] Bonney R. C. Scanlon M. J. Jones D. L. Reed M. J. Anderson M. C. James V. H. (1986). The relationship between oestradiol metabolism and adrenal steroids in the endometrium of postmenopausal women with and without endometrial cancer. Eur. J. Cancer Clin. Oncol. 22, 953–961. 10.1016/0277-5379(86)90062-3 3021469

[B10] Borghese B. Mondon F. Noël J. C. Fayt I. Mignot T. M. Vaiman D.. (2008). Gene expression profile for ectopic versus eutopic endometrium provides new insights into endometriosis oncogenic potential. Mol. Endocrinol. 22, 2557–2562. 10.1210/me.2008-0322 18818281

[B11] Brookes K. J. Hawi Z. Park J. Scott S. Gill M. Kent L. (2010). Polymorphisms of the steroid sulfatase (STS) gene are associated with attention deficit hyperactivity disorder and influence brain tissue mRNA expression. Am. J. Med. Genet. B Neuropsychiatr. Genet. 153B, 1417–1424. 10.1002/ajmg.b.31120 20862695 PMC3132592

[B12] Brosens I. Benagiano G. (2011). Endometriosis, a modern syndrome. Indian J. Med. Res. 133, 581–593. 21727656 PMC3135985

[B13] Bulun S. E. (2013). Uterine fibroids. N. Engl. J. Med. 369, 1344–1355. 10.1056/NEJMra1209993 24088094

[B14] Burney R. O. Talbi S. Hamilton A. E. Vo K. C. Nyegaard M. Nezhat C. R.. (2007). Gene expression analysis of endometrium reveals progesterone resistance and candidate susceptibility genes in women with endometriosis. Endocrinology 148, 3814–3826. 10.1210/en.2006-1692 17510236

[B15] Carlström K. Bergqvist A. Ljungberg O. (1988). Metabolism of estrone sulfate in endometriotic tissue and in uterine endometrium in proliferative and secretory cycle phase. Fertil. Steril. 49, 229–233. 2828122 10.1016/s0015-0282(16)59707-6

[B16] Caron P. Audet-Walsh E. Lépine J. Bélanger A. Guillemette C. (2009). Profiling endogenous serum estrogen and estrogen-glucuronides by liquid chromatography-tandem mass spectrometry. Anal. Chem. 81, 10143–10148. 10.1021/ac9019126 19916521

[B17] Chetrite G. S. Cortes-Prieto J. Philippe J. C. Wright F. Pasqualini J. R. (2000). Comparison of estrogen concentrations, estrone sulfatase and aromatase activities in normal, and in cancerous, human breast tissues. J. Steroid Biochem. Mol. Biol. 72, 23–27. 10.1016/S0960-0760(00)00040-6 10731634

[B18] Choi J. Y. Lee K. M. Park S. K. Noh D. Y. Ahn S. H. Chung H. W.. (2005). Genetic polymorphisms of SULT1A1 and SULT1E1 and the risk and survival of breast cancer. Cancer Epidemiol. Biomarkers Prev. 14, 1090–1095. 10.1158/1055-9965.EPI-04-0688 15894657

[B19] Chura J. C. Blomquist C. H. Ryu H. S. Argenta P. A. (2009). Estrone sulfatase activity in patients with advanced ovarian cancer. Gynecol. Oncol. 112, 205–209. 10.1016/j.ygyno.2008.08.037 18947862

[B20] Colette S. Defrère S. Lousse J. C. Van Langendonckt A. Gotteland J. P. Loumaye E.. (2011). Inhibition of steroid sulfatase decreases endometriosis in an *in vivo* murine model. Hum. Reprod. 26, 1362–1370. 10.1093/humrep/der079 21441545

[B21] Colette S. Defrère S. Van Kerk O. Van Langendonckt A. Dolmans M. M. Donnez J. (2013). Differential expression of steroidogenic enzymes according to endometriosis type. Fertil. Steril. 100, 1642–1649. 10.1016/j.fertnstert.2013.08.003 24012197

[B22] Coughtrie M. W. (2002). Sulfation through the looking glass–recent advances in sulfotransferase research for the curious. Pharmacogenomics J. 2, 297–308. 10.1038/sj.tpj.6500117 12439736

[B23] Dassen H. Punyadeera C. Kamps R. Delvoux B. Van Langendonckt A. Donnez J.. (2007). Estrogen metabolizing enzymes in endometrium and endometriosis. Hum. Reprod. 22, 3148–3158. 10.1093/humrep/dem310 17921479

[B24] Day J. M. Purohit A. Tutill H. J. Foster P. A. Woo L. W. Potter B. V.. (2009). The development of steroid sulfatase inhibitors for hormone-dependent cancer therapy. Ann. N.Y. Acad. Sci. 1155, 80–87. 10.1111/j.1749-6632.2008.03677.x 19250195

[B25] Delvoux B. Groothuis P. D'Hooghe T. Kyama C. Dunselman G. Romano A. (2009). Increased production of 17beta-estradiol in endometriosis lesions is the result of impaired metabolism. J. Clin. Endocrinol. Metab. 94, 876–883. 10.1210/jc.2008-2218 19088158

[B26] Duanmu Z. Kocarek T. Runge-Morris M. (2007). Regulation of human hepatic estrogen sulfotransferase (SULT1E1) expression by xenobiotics and lipid intermediates. FASEB J. 21, 886.19.

[B27] Echchgadda I. Song C. S. Oh T. Ahmed M. De La Cruz I. J. Chatterjee B. (2007). The xenobiotic-sensing nuclear receptors pregnane X receptor, constitutive androstane receptor, and orphan nuclear receptor hepatocyte nuclear factor 4alpha in the regulation of human steroid-/bile acid-sulfotransferase. Mol. Endocrinol. 21, 2099–2111. 10.1210/me.2007-0002 17595319

[B28] Elias P. M. Williams M. L. Choi E. H. Feingold K. R. (2014). Role of cholesterol sulfate in epidermal structure and function: lessons from X-linked ichthyosis. Biochim. Biophys. Acta 1841, 353–361. 10.1016/j.bbalip.2013.11.009 24291327 PMC3966299

[B29] Ezaki K. Motoyama H. Sasaki H. (2001). Immunohistologic localization of estrone sulfatase in uterine endometrium and adenomyosis. Obstet. Gynecol. 98(5 Pt 1), 815–819. 10.1016/S0029-7844(01)01554-X 11704174

[B30] Falany C. N. Krasnykh V. Falany J. L. (1995). Bacterial expression and characterization of a cDNA for human liver estrogen sulfotransferase. J. Steroid Biochem. Mol. Biol. 52, 529–539. 10.1016/0960-0760(95)00015-R 7779757

[B31] Falany J. L. Falany C. N. (1996). Regulation of estrogen sulfotransferase in human endometrial adenocarcinoma cells by progesterone. Endocrinology 137, 1395–1401. 8625916 10.1210/endo.137.4.8625916

[B32] Falany J. L. Falany C. N. (2006). Regulation of SULT1E1 expression in Ishikawa adenocarcinoma cells by tibolone. Steroids 71, 880–885. 10.1016/j.steroids.2006.05.018 16857224

[B33] Fathalla M. F. (2013). Incessant ovulation and ovarian cancer - a hypothesis re-visited. Facts Views Vis. Obgyn. 5, 292–297. 24753957 PMC3987381

[B34] Fechner S. Husen B. Thole H. Schmidt M. Gashaw I. Kimmig R.. (2007). Expression and regulation of estrogen-converting enzymes in ectopic human endometrial tissue. Fertil. Steril. 88(4 Suppl.), 1029–1038. 10.1016/j.fertnstert.2006.11.153 17316633

[B35] Ferlay J. Soerjomatraram I. Ervik M. Dikshit R. Eser S. Mathers C. (2013). GLOBOCAN 2012 v1.0, Cancer Incidence and Mortality Worldwide: IARC CancerBase No. 11 (Internet). Lyon: International Agency for Research on Cancer Available online at: http://globocan.iarc.fr

[B36] Foster P. A. Chander S. K. Newman S. P. Woo L. W. Sutcliffe O. B. Bubert C.. (2008a). A new therapeutic strategy against hormone-dependent breast cancer: the preclinical development of a dual aromatase and sulfatase inhibitor. Clin. Cancer Res. 14, 6469–6477. 10.1158/1078-0432.CCR-08-1027 18927286

[B37] Foster P. A. Chander S. K. Parsons M. F. Newman S. P. Woo L. W. Potter B. V.. (2008b). Efficacy of three potent steroid sulfatase inhibitors: pre-clinical investigations for their use in the treatment of hormone-dependent breast cancer. Breast Cancer Res. Treat. 111, 129–138. 10.1007/s10549-007-9769-3 17914670

[B38] Foster P. A. Woo L. W. Potter B. V. Reed M. J. Purohit A. (2008c). The use of steroid sulfatase inhibitors as a novel therapeutic strategy against hormone-dependent endometrial cancer. Endocrinology 149, 4035–4042. 10.1210/en.2008-0223 18450955 PMC2488239

[B39] Fournier M. A. Poirier D. (2009). Estrogen formation in endometrial and cervix cancer cell lines: involvement of aromatase, steroid sulfatase and 17beta-hydroxysteroid dehydrogenases (types 1, 5, 7 and 12). Mol. Cell. Endocrinol. 301, 142–145. 10.1016/j.mce.2008.08.027 18817841

[B40] Freimuth R. R. Wiepert M. Chute C. G. Wieben E. D. Weinshilboum R. M. (2004). Human cytosolic sulfotransferase database mining: identification of seven novel genes and pseudogenes. Pharmacogenomics J. 4, 54–65. 10.1038/sj.tpj.6500223 14676822

[B41] Fu J. Weise A. M. Falany J. L. Falany C. N. Thibodeau B. J. Miller F. R.. (2010). Expression of estrogenicity genes in a lineage cell culture model of human breast cancer progression. Breast Cancer Res. Treat. 120, 35–45. 10.1007/s10549-009-0363-8 19308726 PMC3772670

[B42] Gamage N. Barnett A. Hempel N. Duggleby R. G. Windmill K. F. Martin J. L.. (2006). Human sulfotransferases and their role in chemical metabolism. Toxicol. Sci. 90, 5–22. 10.1093/toxsci/kfj061 16322073

[B43] Geese W. J. Raftogianis R. B. (2001). Biochemical characterization and tissue distribution of human SULT2B1. Biochem. Biophys. Res. Commun. 288, 280–289. 10.1006/bbrc.2001.5746 11594786

[B44] Gelmetti C. Ruggero C. (2002). Pediatric Dermatology and Dermatopathology: A Concise Atlas. London: T&F STM.

[B45] Ghosh D. (2007). Human sulfatases: a structural perspective to catalysis. Cell. Mol. Life Sci. 64, 2013–2022. 10.1007/s00018-007-7175-y 17558559 PMC11136368

[B46] Gosavi R. A. Knudsen G. A. Birnbaum L. S. Pedersen L. C. (2013). Mimicking of estradiol binding by flame retardants and their metabolites: a crystallographic analysis. Environ. Health Perspect. 121, 1194–1199. 10.1289/ehp.1306902 23959441 PMC3801471

[B47] Graziano A. Lo Monte G. Piva I. Caserta D. Karner M. Engl B.. (2015). Diagnostic findings in adenomyosis: a pictorial review on the major concerns. Eur. Rev. Med. Pharmacol. Sci. 19, 1146–1154. 25912572

[B48] Guarneri V. Barbieri E. Dieci M. V. Piacentini F. Conte P. (2011). Timing for starting second-line therapy in recurrent ovarian cancer. Expert Rev. Anticancer Ther. 11, 49–55. 10.1586/era.10.204 21166510

[B49] Guo S. W. Wang Y. (2006). Sources of heterogeneities in estimating the prevalence of endometriosis in infertile and previously fertile women. Fertil. Steril. 86, 1584–1595. 10.1016/j.fertnstert.2006.04.040 17067588

[B50] Henderson B. E. Feigelson H. S. (2000). Hormonal carcinogenesis. Carcinogenesis 21, 427–433. 10.1093/carcin/21.3.427 10688862

[B51] Hernandez-Guzman F. G. Higashiyama T. Osawa Y. Ghosh D. (2001). Purification, characterization and crystallization of human placental estrone/dehydroepiandrosterone sulfatase, a membrane-bound enzyme of the endoplasmic reticulum. J. Steroid Biochem. Mol. Biol. 78, 441–450. 10.1016/S0960-0760(01)00119-4 11738554

[B52] Hernandez-Guzman F. G. Higashiyama T. Pangborn W. Osawa Y. Ghosh D. (2003). Structure of human estrone sulfatase suggests functional roles of membrane association. J. Biol. Chem. 278, 22989–22997. 10.1074/jbc.M211497200 12657638

[B53] Hevir N. Ribic-Pucelj M. Lanišnik Rižner T. (2013). Disturbed balance between phase I and II metabolizing enzymes in ovarian endometriosis: a source of excessive hydroxy-estrogens and ROS? Mol. Cell. Endocrinol. 367, 74–84. 10.1016/j.mce.2012.12.019 23277161

[B54] Hevir N. Sinkovec J. Rižner T. L. (2011). Disturbed expression of phase I and phase II estrogen-metabolizing enzymes in endometrial cancer: lower levels of CYP1B1 and increased expression of S-COMT. Mol. Cell. Endocrinol. 331, 158–167. 10.1016/j.mce.2010.09.011 20887769

[B55] Hevir-Kene N. Rižner T. L. (2015). The endometrial cancer cell lines Ishikawa and HEC-1A, and the control cell line HIEEC, differ in expression of estrogen biosynthetic and metabolic genes, and in androstenedione and estrone-sulfate metabolism. Chem. Biol. Interact. 234, 309–319. 10.1016/j.cbi.2014.11.015 25437045

[B56] Hildebrandt M. A. Carrington D. P. Thomae B. A. Eckloff B. W. Schaid D. J. Yee V. C.. (2007). Genetic diversity and function in the human cytosolic sulfotransferases. Pharmacogenomics J. 7, 133–143. 10.1038/sj.tpj.6500404 16801938

[B57] Hirata H. Hinoda Y. Okayama N. Suehiro Y. Kawamoto K. Kikuno N.. (2008). CYP1A1, SULT1A1, and SULT1E1 polymorphisms are risk factors for endometrial cancer susceptibility. Cancer 112, 1964–1973. 10.1002/cncr.23392 18318428

[B58] Ho Y. T. Purohit A. Vicker N. Newman S. P. Robinson J. J. Leese M. P.. (2003). Inhibition of carbonic anhydrase II by steroidal and non-steroidal sulphamates. Biochem. Biophys. Res. Commun. 305, 909–914. 10.1016/S0006-291X(03)00865-9 12767917

[B59] Hudelist G. Czerwenka K. Keckstein J. Haas C. Fink-Retter A. Gschwantler-Kaulich D.. (2007). Expression of aromatase and estrogen sulfotransferase in eutopic and ectopic endometrium: evidence for unbalanced estradiol production in endometriosis. Reprod. Sci. 14, 798–805. 10.1177/1933719107309120 18089598

[B60] Huhtinen K. Suvitie P. Hiissa J. Junnila J. Huvila J. Kujari H.. (2009). Serum HE4 concentration differentiates malignant ovarian tumours from ovarian endometriotic cysts. Br. J. Cancer 100, 1315–1319. 10.1038/sj.bjc.6605011 19337252 PMC2676558

[B61] Inoue M. (2001). Current molecular aspects of the carcinogenesis of the uterine endometrium. Int. J. Gynecol. Cancer 11, 339–348. 10.1046/j.1525-1438.2001.01046.x 11737463

[B62] Ishida H. Nakata T. Suzuki M. Shiotsu Y. Tanaka H. Sato N.. (2007). A novel steroidal selective steroid sulfatase inhibitor KW-2581 inhibits sulfated-estrogen dependent growth of breast cancer cells *in vitro* and in animal models. Breast Cancer Res. Treat. 106, 215–227. 10.1007/s10549-007-9495-x 17268815

[B63] Jasonni V. M. Bulletti C. Franceschetti F. Bonavia M. Bolelli G. Ciotti P.. (1984). Estrone sulphate plasma levels in postmenopausal women with and without endometrial cancer. Cancer 53, 2698–2700. 6722729 10.1002/1097-0142(19840615)53:12<2698::aid-cncr2820531223>3.0.co;2-o

[B64] Kent L. Emerton J. Bhadravathi V. Weisblatt E. Pasco G. Willatt L. R.. (2008). X-linked ichthyosis (steroid sulfatase deficiency) is associated with increased risk of attention deficit hyperactivity disorder, autism and social communication deficits. J. Med. Genet. 45, 519–524. 10.1136/jmg.2008.057729 18413370

[B65] Kester M. H. Bulduk S. Tibboel D. Meinl W. Glatt H. Falany C. N.. (2000). Potent inhibition of estrogen sulfotransferase by hydroxylated PCB metabolites: a novel pathway explaining the estrogenic activity of PCBs. Endocrinology 141, 1897–1900. 10.1210/endo.141.5.7530 10803601

[B66] Kitawaki J. (2006). Adenomyosis: the pathophysiology of an oestrogen-dependent disease. Best Pract. Res. Clin. Obstet. Gynaecol. 20, 493–502. 10.1016/j.bpobgyn.2006.01.010 16564227

[B67] Kitawaki J. Noguchi T. Amatsu T. Maeda K. Tsukamoto K. Yamamoto T.. (1997). Expression of aromatase cytochrome P450 protein and messenger ribonucleic acid in human endometriotic and adenomyotic tissues but not in normal endometrium. Biol. Reprod. 57, 514–519. 10.1095/biolreprod57.3.514 9282984

[B68] Kästingschäfer C. S. Schäfer S. D. Kiesel L. Götte M. (2015). miR-142-3p is a novel regulator of cell viability and proinflammatory signalling in endometrial stroma cells. Reprod. Biomed. Online 30, 553–556. 10.1016/j.rbmo.2015.01.002 25754227

[B69] Koizumi M. Momoeda M. Hiroi H. Hosokawa Y. Tsutsumi R. Osuga Y.. (2010). Expression and regulation of cholesterol sulfotransferase (SULT2B1b) in human endometrium. Fertil. Steril. 93, 1538–1544. 10.1016/j.fertnstert.2009.01.075 19243756

[B70] Labrie F. (1991). Intracrinology. Mol. Cell. Endocrinol. 78, C113–C118. 10.1016/0303-7207(91)90116-A 1838082

[B71] Labrie F. Bélanger A. Bélanger P. Bérubé R. Martel C. Cusan L.. (2006). Androgen glucuronides, instead of testosterone, as the new markers of androgenic activity in women. J. Steroid Biochem. Mol. Biol. 99, 182–188. 10.1016/j.jsbmb.2006.02.004 16621522

[B72] Labrie F. Cusan L. Gomez J. L. Martel C. Bérubé R. Bélanger P.. (2009). Comparable amounts of sex steroids are made outside the gonads in men and women: strong lesson for hormone therapy of prostate and breast cancer. J. Steroid Biochem. Mol. Biol. 113, 52–56. 10.1016/j.jsbmb.2008.11.004 19073258

[B73] Lee K. A. Fuda H. Lee Y. C. Negishi M. Strott C. A. Pedersen L. C. (2003). Crystal structure of human cholesterol sulfotransferase (SULT2B1b) in the presence of pregnenolone and 3′-phosphoadenosine 5′-phosphate. Rationale for specificity differences between prototypical SULT2A1 and the SULT2BG1 isoforms. J. Biol. Chem. 278, 44593–44599. 10.1074/jbc.M308312200 12923182

[B74] Li W. Ning M. Koh K. H. Kim H. Jeong H. (2014). 17β-Estradiol induces sulfotransferase 2A1 expression through estrogen receptor α. Drug Metab. Dispos. 42, 796–802. 10.1124/dmd.113.055178 24492894 PMC3965899

[B75] Lindsay J. Wang L. L. Li Y. Zhou S. F. (2008). Structure, function and polymorphism of human cytosolic sulfotransferases. Curr. Drug Metab. 9, 99–105. 10.2174/138920008783571819 18288952

[B76] Lu L. Y. Hsieh Y. C. Liu M. Y. Lin Y. H. Chen C. J. Yang Y. S. (2008). Identification and characterization of two amino acids critical for the substrate inhibition of human dehydroepiandrosterone sulfotransferase (SULT2A1). Mol. Pharmacol. 73, 660–668. 10.1124/mol.107.041038 18042734

[B77] Lurie G. Wilkens L. R. Thompson P. J. Shvetsov Y. B. Matsuno R. K. Carney M. E.. (2011). Estrogen receptor beta rs1271572 polymorphism and invasive ovarian carcinoma risk: pooled analysis within the Ovarian Cancer Association Consortium. PLoS ONE 6:e20703. 10.1371/journal.pone.0020703 21673961 PMC3108970

[B78] Luu-The V. Dufort I. Paquet N. Reimnitz G. Labrie F. (1995). Structural characterization and expression of the human dehydroepiandrosterone sulfotransferase gene. DNA Cell Biol. 14, 511–518. 10.1089/dna.1995.14.511 7598806

[B79] Lépine J. Audet-Walsh E. Grégoire J. Têtu B. Plante M. Ménard V.. (2010). Circulating estrogens in endometrial cancer cases and their relationship with tissular expression of key estrogen biosynthesis and metabolic pathways. J. Clin. Endocrinol. Metab. 95, 2689–2698. 10.1210/jc.2010-2648 20371658

[B80] Lévesque E. Laverdière I. Audet-Walsh E. Caron P. Rouleau M. Fradet Y.. (2014). Steroidogenic germline polymorphism predictors of prostate cancer progression in the estradiol pathway. Clin. Cancer Res. 20, 2971–2983. 10.1158/1078-0432.CCR-13-2567 24682418

[B81] Maitoko K. Sasaki H. (2004). Gonadotropin-releasing hormone agonist inhibits estrone sulfatase expression of cystic endometriosis in the ovary. Fertil. Steril. 82, 322–326. 10.1016/j.fertnstert.2003.12.044 15302278

[B82] Maltais R. Poirier D. (2011). Steroid sulfatase inhibitors: a review covering the promising 2000-2010 decade. Steroids 76, 929–948. 10.1016/j.steroids.2011.03.010 21458474

[B83] Matsumoto J. Ariyoshi N. Ishii I. Kitada M. (2010). Six novel single nucleotide polymorphisms of the steroid sulfatase gene in a Japanese population. Drug Metab. Pharmacokinet. 25, 403–407. 10.2133/dmpk.DMPK-10-SC-027 20814163

[B84] Matsumoto J. Ariyoshi N. Ishii I. Kitada M. (2013). Functional characterization of seven single-nucleotide polymorphisms of the steroid sulfatase gene found in a Japanese population. J. Hum. Genet. 58, 267–272. 10.1038/jhg.2013.12 23466819

[B85] Matsuoka R. Yanaihara A. Saito H. Furusawa Y. Toma Y. Shimizu Y.. (2002). Regulation of estrogen activity in human endometrium: effect of IL-1beta on steroid sulfatase activity in human endometrial stromal cells. Steroids 67, 655–659. 10.1016/S0039-128X(02)00016-8 11996939

[B86] Meloche C. A. Falany C. N. (2001). Expression and characterization of the human 3 beta-hydroxysteroid sulfotransferases (SULT2B1a and SULT2B1b). J. Steroid Biochem. Mol. Biol. 77, 261–269. 10.1016/S0960-0760(01)00064-4 11457664

[B87] Modugno F. Laskey R. Smith A. L. Andersen C. L. Haluska P. Oesterreich S. (2012). Hormone response in ovarian cancer: time to reconsider as a clinical target? Endocr. Relat. Cancer 19, R255–R279. 10.1530/ERC-12-0175 23045324 PMC3696394

[B88] Moravek M. B. Yin P. Ono M. Coon J. S. Dyson M. T. Navarro A.. (2015). Ovarian steroids, stem cells and uterine leiomyoma: therapeutic implications. Hum. Reprod. Update 21, 1–12. 10.1093/humupd/dmu048 25205766 PMC4255606

[B89] Mostafa Y. A. Taylor S. D. (2013). Steroid derivatives as inhibitors of steroid sulfatase. J. Steroid Biochem. Mol. Biol. 137, 183–198. 10.1016/j.jsbmb.2013.01.013 23391659

[B90] Mueller J. W. Gilligan L. C. Idkowiak J. Arlt W. Foster P. A. (2015). The regulation of steroid action by sulfation and desulfation. Endocr. Rev. 36, 526–563. 10.1210/er.2015-1036 26213785 PMC4591525

[B91] Nagata K. Yamazoe Y. (2000). Pharmacogenetics of sulfotransferase. Annu. Rev. Pharmacol. Toxicol. 40, 159–176. 10.1146/annurev.pharmtox.40.1.159 10836131

[B92] Naitoh K. Honjo H. Yamamoto T. Urabe M. Ogino Y. Yasumura T.. (1989). Estrone sulfate and sulfatase activity in human breast cancer and endometrial cancer. J. Steroid Biochem. 33, 1049–1054. 10.1016/0022-4731(89)90408-1 2559248

[B93] Nardi A. Pomari E. Zambon D. Belvedere P. Colombo L. Dalla Valle L. (2009). Transcriptional control of human steroid sulfatase. J. Steroid Biochem. Mol. Biol. 115, 68–74. 10.1016/j.jsbmb.2009.02.017 19429462

[B94] Negishi M. Pedersen L. G. Petrotchenko E. Shevtsov S. Gorokhov A. Kakuta Y.. (2001). Structure and function of sulfotransferases. Arch. Biochem. Biophys. 390, 149–157. 10.1006/abbi.2001.2368 11396917

[B95] Newman S. P. Purohit A. Ghilchik M. W. Potter B. V. Reed M. J. (2000). Regulation of steroid sulphatase expression and activity in breast cancer. J. Steroid Biochem. Mol. Biol. 75, 259–264. 10.1016/S0960-0760(00)00177-1 11282280

[B96] Okuda T. Saito H. Sekizawa A. Shimizu Y. Akamatsu T. Kushima M.. (2001). Steroid sulfatase expression in ovarian clear cell adenocarcinoma: immunohistochemical study. Gynecol. Oncol. 82, 427–434. 10.1006/gyno.2001.6322 11520136

[B97] Pasqualini J. R. (2009). Estrogen sulfotransferases in breast and endometrial cancers. Ann. N.Y. Acad. Sci. 1155, 88–98. 10.1111/j.1749-6632.2009.04113.x 19250196

[B98] Pasqualini J. R. Chetrite G. Nestour E. L. (1996). Control and expression of oestrone sulphatase activities in human breast cancer. J. Endocrinol. 150(Suppl.), S99–S105. 8943793

[B99] Pasqualini J. R. Cornier E. Grenier J. Vella C. Schatz B. Netter A. (1990). Effect of decapeptyl (D-TrpG GnPh) on estrogen receptors, progesterone receptors and tissue levels of estrogens (non-conjugated and sulfate-conjugated) in patients with uterine myoma. Pathol. Biol. 38, 941–943. 2148976

[B100] Pautier P. Lobbedez F. Melchar B. Kutarska E. Hall G. Reed N. (2012). A phase II multicentre randomized open-label study of oral steroid sulphatase (STS) inhibitor irosustat (BN83495) versus megestrol acetate (MA) in women with advanced/recurrent endometrial cancer. Annals Oncol. 23(suppl. 9), 329.

[B101] Pedersen L. C. Petrotchenko E. Shevtsov S. Negishi M. (2002). Crystal structure of the human estrogen sulfotransferase-PAPS complex: evidence for catalytic role of Ser137 in the sulfuryl transfer reaction. J. Biol. Chem. 277, 17928–17932. 10.1074/jbc.M111651200 11884392

[B102] Pohl O. Bestel E. Gotteland J. P. (2014). Synergistic effects of E2MATE and norethindrone acetate on steroid sulfatase inhibition: a randomized phase I proof-of-principle clinical study in women of reproductive age. Reprod. Sci. 21, 1256–1265. 10.1177/1933719114522526 24604234

[B103] Poirier D. (2015). Recent patents on new steroid agents targeting the steroidogenesis for endocrine cancer treatments. Recent Pat. Endocr. Metab. Immune Drug Discov. 9, 15–23. 10.2174/1872214809666150201214602 25643356

[B104] Preusser-Kunze A. Mariappan M. Schmidt B. Gande S. L. Mutenda K. Wenzel D.. (2005). Molecular characterization of the human Calpha-formylglycine-generating enzyme. J. Biol. Chem. 280, 14900–14910. 10.1074/jbc.M413383200 15657036

[B105] Prost O. Turrel M. O. Dahan N. Craveur C. Adessi G. L. (1984). Estrone and dehydroepiandrosterone sulfatase activities and plasma estrone sulfate levels in human breast carcinoma. Cancer Res. 44, 661–664. 6581863

[B106] Purohit A. Fusi L. Brosens J. Woo L. W. Potter B. V. Reed M. J. (2008). Inhibition of steroid sulphatase activity in endometriotic implants by 667 COUMATE: a potential new therapy. Hum. Reprod. 23, 290–297. 10.1093/humrep/dem308 18056119

[B107] Purohit A. Potter B. V. Parker M. G. Reed M. J. (1998). Steroid sulphatase: expression, isolation and inhibition for active-site identification studies. Chem. Biol. Interact. 109, 183–193. 10.1016/S0009-2797(97)00132-4 9566745

[B108] Purohit A. Woo L. W. Potter B. V. (2011). Steroid sulfatase: a pivotal player in estrogen synthesis and metabolism. Mol. Cell. Endocrinol. 340, 154–160. 10.1016/j.mce.2011.06.012 21693170

[B109] Raftogianis R. Creveling C. Weinshilboum R. Weisz J. (2000). Estrogen metabolism by conjugation. J. Natl. Cancer Inst. Monogr. 27, 113–124. 10.1093/oxfordjournals.jncimonographs.a024234 10963623

[B110] Rasmussen L. M. Zaveri N. T. Stenvang J. Peters R. H. Lykkesfeldt A. E. (2007). A novel dual-target steroid sulfatase inhibitor and antiestrogen: SR 16157, a promising agent for the therapy of breast cancer. Breast Cancer Res. Treat. 106, 191–203. 10.1007/s10549-007-9494-y 17268816

[B111] Rebbeck T. R. Troxel A. B. Wang Y. Walker A. H. Panossian S. Gallagher S.. (2006). Estrogen sulfation genes, hormone replacement therapy, and endometrial cancer risk. J. Natl. Cancer Inst. 98, 1311–1320. 10.1093/jnci/djj360 16985250

[B112] Reed M. J. Purohit A. Woo L. W. Newman S. P. Potter B. V. (2005). Steroid sulfatase: molecular biology, regulation, and inhibition. Endocr. Rev. 26, 171–202. 10.1210/er.2004-0003 15561802

[B113] Rehse P. H. Zhou M. Lin S. X. (2002). Crystal structure of human dehydroepiandrosterone sulphotransferase in complex with substrate. Biochem. J. 364(Pt 1), 165–171. 10.1042/bj3640165 11988089 PMC1222558

[B114] Ren X. Wu X. Hillier S. G. Fegan K. S. Critchley H. O. Mason J. I.. (2015). Local estrogen metabolism in epithelial ovarian cancer suggests novel targets for therapy. J. Steroid Biochem. Mol. Biol. 150, 54–63. 10.1016/j.jsbmb.2015.03.010 25817828 PMC4429663

[B115] Rižner T. L. (2009). Estrogen metabolism and action in endometriosis. Mol. Cell. Endocrinol. 307, 8–18. 10.1016/j.mce.2009.03.022 19524121

[B116] Rižner T. L. (2013). Estrogen biosynthesis, phase I and phase II metabolism, and action in endometrial cancer. Mol. Cell. Endocrinol. 381, 124–139. 10.1016/j.mce.2013.07.026 23911898

[B117] Rogers P. A. D'Hooghe T. M. Fazleabas A. Giudice L. C. Montgomery G. W. Petraglia F.. (2013). Defining future directions for endometriosis research: workshop report from the 2011 World Congress of Endometriosis In Montpellier, France. Reprod. Sci. 20, 483–499. 10.1177/1933719113477495 23427182 PMC3635070

[B118] Rubin G. L. Harrold A. J. Mills J. A. Falany C. N. Coughtrie M. W. (1999). Regulation of sulphotransferase expression in the endometrium during the menstrual cycle, by oral contraceptives and during early pregnancy. Mol. Hum. Reprod. 5, 995–1002. 10.1093/molehr/5.11.995 10541560

[B119] Ryan A. J. Susil B. Jobling T. W. Oehler M. K. (2005). Endometrial cancer. Cell Tissue Res. 322, 53–61. 10.1007/s00441-005-1109-5 15947972

[B120] Sadozai H. (2013). Steroid sulfatase inhibitors: promising new therapy for breast cancer. J. Pak. Med. Assoc. 63, 509–515. 23905452

[B121] Saner K. J. Suzuki T. Sasano H. Pizzey J. Ho C. Strauss J. F.. (2005). Steroid sulfotransferase 2A1 gene transcription is regulated by steroidogenic factor 1 and GATA-6 in the human adrenal. Mol. Endocrinol. 19, 184–197. 10.1210/me.2003-0332 15388788

[B122] Seo Y. K. Mirkheshti N. Song C. S. Kim S. Dodds S. Ahn S. C.. (2013). SULT2B1b sulfotransferase: induction by vitamin D receptor and reduced expression in prostate cancer. Mol. Endocrinol. 27, 925–939. 10.1210/me.2012-1369 23579488 PMC3656233

[B123] Simpson E. R. (2003). Sources of estrogen and their importance. J. Steroid Biochem. Mol. Biol. 86, 225–230. 10.1016/S0960-0760(03)00360-1 14623515

[B124] Smuc T. Hevir N. Ribic-Pucelj M. Husen B. Thole H. Rizner T. L. (2009). Disturbed estrogen and progesterone action in ovarian endometriosis. Mol. Cell. Endocrinol. 301, 59–64. 10.1016/j.mce.2008.07.020 18762229

[B125] Smuc T. Pucelj M. R. Sinkovec J. Husen B. Thole H. Lanisnik Rizner T. (2007). Expression analysis of the genes involved in estradiol and progesterone action in human ovarian endometriosis. Gynecol. Endocrinol. 23, 105–111. 10.1080/09513590601152219 17454161

[B126] Smuc T. Rizner T. L. (2009). Aberrant pre-receptor regulation of estrogen and progesterone action in endometrial cancer. Mol. Cell. Endocrinol. 301, 74–82. 10.1016/j.mce.2008.09.019 18930784

[B127] Sonoda Y. Barakat R. R. (2006). Screening and the prevention of gynecologic cancer: endometrial cancer. Best Pract. Res. Clin. Obstet. Gynaecol. 20, 363–377. 10.1016/j.bpobgyn.2005.10.015 16364689

[B128] Stadel B. V. (1975). Letter: the etiology and prevention of ovarian cancer. Am. J. Obstet. Gynecol. 123, 772–774. 1200073 10.1016/0002-9378(75)90509-8

[B129] Stanway S. J. Purohit A. Woo L. W. Sufi S. Vigushin D. Ward R.. (2006). Phase I study of STX 64 (667 Coumate) in breast cancer patients: the first study of a steroid sulfatase inhibitor. Clin. Cancer Res. 12, 1585–1592. 10.1158/1078-0432.CCR-05-1996 16533785

[B130] Stergiakouli E. Langley K. Williams H. Walters J. Williams N. M. Suren S.. (2011). Steroid sulfatase is a potential modifier of cognition in attention deficit hyperactivity disorder. Genes Brain Behav. 10, 334–344. 10.1111/j.1601-183X.2010.00672.x 21255266 PMC3664024

[B131] Sung C. H. Im H. J. Park N. Kwon Y. Shin S. Ye D. J.. (2013). Induction of steroid sulfatase expression in PC-3 human prostate cancer cells by insulin-like growth factor II. Toxicol. Lett. 223, 109–115. 10.1016/j.toxlet.2013.09.006 24055520

[B132] Tanaka K. Kubushiro K. Iwamori Y. Okairi Y. Kiguchi K. Ishiwata I.. (2003). Estrogen sulfotransferase and sulfatase: roles in the regulation of estrogen activity in human uterine endometrial carcinomas. Cancer Sci. 94, 871–876. 10.1111/j.1349-7006.2003.tb01369.x 14556660 PMC11160014

[B133] Thomae B. A. Eckloff B. W. Freimuth R. R. Wieben E. D. Weinshilboum R. M. (2002). Human sulfotransferase SULT2A1 pharmacogenetics: genotype-to-phenotype studies. Pharmacogenomics J. 2, 48–56. 10.1038/sj.tpj.6500089 11990382

[B134] Thomas M. P. Potter B. V. (2013). The structural biology of oestrogen metabolism. J. Steroid Biochem. Mol. Biol. 137, 27–49. 10.1016/j.jsbmb.2012.12.014 23291110 PMC3866684

[B135] Thomas M. P. Potter B. V. (2015). Estrogen O-sulfamates and their analogues: clinical steroid sulfatase inhibitors with broad potential. J. Steroid Biochem. Mol. Biol. 153, 160–169. 10.1016/j.jsbmb.2015.03.012 25843211

[B136] Tibbs Z. E. Rohn-Glowacki K. J. Crittenden F. Guidry A. L. Falany C. N. (2015). Structural plasticity in the human cytosolic sulfotransferase dimer and its role in substrate selectivity and catalysis. Drug Metab. Pharmacokinet. 30, 3–20. 10.1016/j.dmpk.2014.10.004 25760527

[B137] Tseng L. Mazella J. Mann W. J. Chumas J. (1982). Estrogen synthesis in normal and malignant human endometrium. J. Clin. Endocrinol. Metab. 55, 1029–1031. 10.1210/jcem-55-5-1029 7119084

[B138] Tworoger S. S. Zhang X. Eliassen A. H. Qian J. Colditz G. A. Willett W. C.. (2014). Inclusion of endogenous hormone levels in risk prediction models of postmenopausal breast cancer. J. Clin. Oncol. 32, 3111–3117. 10.1200/JCO.2014.56.1068 25135988 PMC4171356

[B139] Uhlén M. Fagerberg L. Hallström B. M. Lindskog C. Oksvold P. Mardinoglu A.. (2015). Proteomics. Tissue-based map of the human proteome. Science 347:1260419. 10.1126/science.1260419 25613900

[B140] Urabe M. Yamamoto T. Naitoh K. Honjo H. Okada H. (1989). Estrone sulfatase activity in normal and neoplastic endometrial tissues of human uterus. Asia Oceania J. Obstet. Gynaecol. 15, 101–106. 10.1111/j.1447-0756.1989.tb00160.x 2525375

[B141] Utsunomiya H. Ito K. Suzuki T. Kitamura T. Kaneko C. Nakata T.. (2004). Steroid sulfatase and estrogen sulfotransferase in human endometrial carcinoma. Clin. Cancer Res. 10, 5850–5856. 10.1158/1078-0432.CCR-04-0040 15355916

[B142] van de Ven J. Donker T. H. Blankenstein M. A. Thijssen J. H. (2002). Differential effect of gonadotropin-releasing hormone analogue treatment on estrogen levels and sulfatase activity in uterine leiomyoma and myometrium. Fertil. Steril. 77, 1227–1232. 10.1016/S0015-0282(02)03093-5 12057733

[B143] Vaughan S. Coward J. I. Bast R. C. Berchuck A. Berek J. S. Brenton J. D.. (2011). Rethinking ovarian cancer: recommendations for improving outcomes. Nat. Rev. Cancer 11, 719–725. 10.1038/nrc3144 21941283 PMC3380637

[B144] Wang T. Cook I. Falany C. N. Leyh T. S. (2014). Paradigms of sulfotransferase catalysis: the mechanism of SULT2A1. J. Biol. Chem. 289, 26474–26480. 10.1074/jbc.M114.573501 25056952 PMC4176245

[B145] Williams S. J. (2013). Sulfatase inhibitors: a patent review. Expert Opin. Ther. Pat. 23, 79–98. 10.1517/13543776.2013.736965 23136854

[B146] Woo L. L. Purohit A. Malini B. Reed M. J. Potter B. V. (2000). Potent active site-directed inhibition of steroid sulphatase by tricyclic coumarin-based sulphamates. Chem. Biol. 7, 773–791. 10.1016/S1074-5521(00)00023-5 11033081

[B147] Xu Y. Liu X. Guo F. Ning Y. Zhi X. Wang X.. (2012). Effect of estrogen sulfation by SULT1E1 and PAPSS on the development of estrogen-dependent cancers. Cancer Sci. 103, 1000–1009. 10.1111/j.1349-7006.2012.02258.x 22380844 PMC7685083

[B148] Yamamoto T. Noguchi T. Tamura T. Kitawaki J. Okada H. (1993). Evidence for estrogen synthesis in adenomyotic tissues. Am. J. Obstet. Gynecol. 169, 734–738. 10.1016/0002-9378(93)90654-2 8372890

[B149] Yamamoto T. Urabe M. Naitoh K. Kitawaki J. Honjo H. Okada H. (1990). Estrone sulfatase activity in human uterine leiomyoma. Gynecol. Oncol. 37, 315–318. 10.1016/0090-8258(90)90358-R 2351313

[B150] Yeramian A. Moreno-Bueno G. Dolcet X. Catasus L. Abal M. Colas E.. (2013). Endometrial carcinoma: molecular alterations involved in tumor development and progression. Oncogene 32, 403–413. 10.1038/onc.2012.76 22430211

[B151] Zaichuk T. Ivancic D. Scholtens D. Schiller C. Khan S. A. (2007). Tissue-specific transcripts of human steroid sulfatase are under control of estrogen signaling pathways in breast carcinoma. J. Steroid Biochem. Mol. Biol. 105, 76–84. 10.1016/j.jsbmb.2006.12.101 17596930

